# Westward migration of tropical cyclone rapid-intensification over the Northwestern Pacific during short duration El Niño

**DOI:** 10.1038/s41467-018-03945-y

**Published:** 2018-04-17

**Authors:** Yi-Peng Guo, Zhe-Min Tan

**Affiliations:** Key Laboratory of Mesoscale Severe Weather/Ministry of Education and School of Atmospheric Sciences, Nanjing University, Nanjing, 210023 China

## Abstract

The El Niño-Southern Oscillation (ENSO) can significantly affect the rapid intensification of tropical cyclones over the western North Pacific (WNP). However, ENSO events have various durations, which can lead to different atmospheric and oceanic conditions. Here we show that during short duration El Niño events, the WNP tropical cyclone rapid-intensification mean occurrence position migrates westward by ~8.0° longitude, which is caused by reduced vertical wind shear, increased mid-tropospheric humidity, and enhanced tropical cyclone heat potential over the westernmost WNP. The changes in these factors are caused by westward advected upper ocean heat during the decaying phase of a short duration El Niño. As super El Niño events tend to have short durations and their frequency is projected to increase under global warming, our findings have important implications for future projections of WNP tropical cyclone activity.

## Abstract (web summary)

El Niño events have important impacts on tropical cyclone activity over the western North Pacific. Here, the authors show that short duration El Niño could significantly shift the tropical cyclone rapid intensification westward by transporting ocean heat energy to the western Pacific.

## Introduction

Tropical cyclones (TCs) have the potential to cause severe damage to the economy, infrastructure, and inhabitants in affected areas^1–3^. As western North Pacific (WNP) TCs account for nearly one-third of total TCs globally each year, it is important to understand the WNP TC variability as well as its underlying mechanisms. The El Niño-Southern Oscillation (ENSO) modulates many aspects of the WNP TC activity^4–6^. Previous studies have found a close relationship between TC activity and changes in ENSO-related atmospheric environmental parameters, such as the vertical wind shear (VWS), relative humidity (RH), and relative vorticity^4–6^. Although these studies have improved the predictions of TC tracks, forecasting TC intensity change, especially TC rapid intensification (RI), remains challenging^7–9^. ENSO events are important contributors to the WNP TC RI because they affect both large-scale atmospheric circulation and oceanic processes^8^. The large-scale atmospheric circulation associated with ENSO events can lead to meridional shifts in the RI TC (TC with RI process) genesis positions^8^, whereas ENSO-induced upper ocean heat content (OHC) is an important contributor to TC RI over both of the eastern Pacific and the Atlantic^7,10,11^. A recent study revealed that the ocean heat meridionally discharged by ENSO can significantly increase the TC frequency and intensity over the eastern North Pacific^12^. The WNP subsurface OHC is also affected by ENSO^13^, however, the relative roles of ENSO-related changes in the large-scale atmospheric circulation and OHC in affecting the WNP TC RI remain unclear.

El Niño events, which are the warm phases of ENSO, can be categorized into two subtypes based on their duration: short duration (SD) and long duration (LD)^14,15^. SD El Niño events decay rapidly into La Niña events after reaching their peak intensity, whereas LD El Niño events extend to the end of the decaying year with sea surface temperature (SST) anomalies remaining positive over the middle Pacific. Several mechanisms have been proposed to explain the termination of an El Niño event such as the western boundary reflection of Rossby waves^16^, the Ekman transport under local circulation of the western Pacific anticyclone^17^, and the recharge–discharge oscillator^18^. These mechanisms capture the important role of the upper OHC change in determining an El Niño’s duration^14^. However, previous studies have not distinguished between SD and LD El Niño events when exploring the relationship between the El Niño and WNP TC RI. Whether the El Niño event types have different impacts on the WNP TC RI and the role of upper OHC change in affecting TC RI remain unsolved problems. By using multiple atmospheric and oceanic datasets, we found that the WNP TC RI shows an obvious westward migration during the SD El Niño decaying phase due to the westward advection of subsurface OHC.

## Results

### Westward migration of tropical cyclone rapid intensification

**Fig. 1:**
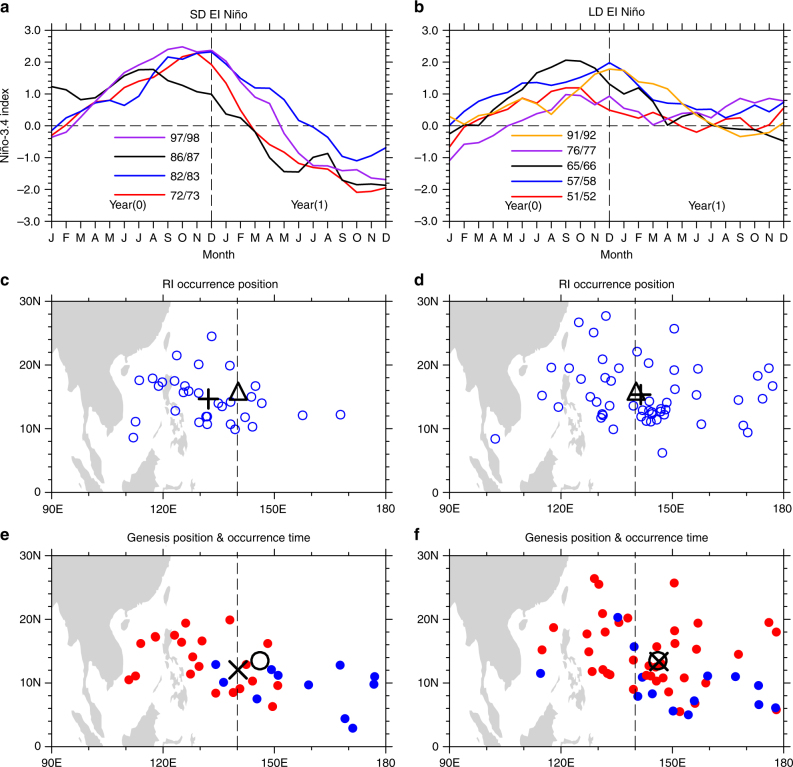
El Niño durations and their impacts on the tropical cyclone rapid intensification. The time series of monthly mean Niño-3.4 indices based on ERSST reanalysis dataset from January of the El Niño developing year to December of the decaying year for **a** short duration (SD) El Niño events and **b** long duration (LD) El Niño events. El Niño developing and decaying years are denoted by year(0) and year(1), respectively. The composite tropical cyclone (TC) rapid intensification (RI) occurrence positions from JTWC best-track dataset for **c** SD and **d** LD El Niño events are shown as blue circles. The triangles in **c**,** d** indicate the mean RI occurrence position for neutral years. The crosses in **c**,** d** indicate the mean RI occurrence positions for SD and LD El Niño events, respectively. The composite RI TC genesis positions for **e** SD and **f** LD El Niño events are shown as colored dots. The red (blue) dots indicate the RI occurrence time is within (beyond) 3 days of TC genesis. The circles in **e**, **f** indicate the mean RI TC genesis position for neutral years. The x-marks in **e**, **f** indicate the mean RI TC genesis position for SD and LD El Niño events, respectively. The dashed lines in **c**–**f** indicate the 140°E longitude

The two types of El Niño events have distinct evolutionary features during their decaying phases. The Niño-3.4 indices for the four SD El Niño events (see Methods section) enter negative phases during summer and autumn, which indicates that these El Niño events are of SD and followed by La Niña events (Fig. 1a), whereas the Niño-3.4 indices for the five LD El Niño events approach zero without entering a negative phase (Fig. 1b). In this study, given the different evolutionary features of SD and LD El Niño events, the focus will be on the different impacts of SD and LD El Niño events on the WNP TC RI during decaying phases. The decaying phase of LD El Niño events can last longer than 1 year, which is not shown in Fig. 1, and the period from July to November (JASON) during the first decaying year of El Niño will be the focus of this study, unless stated otherwise. Without distinguishing the SD and LD El Niño events, the composite difference of the most variables related to WNP TC RI are not statistically significant between the El Niño years and the neutral years (Supplementary Table 1). During an SD El Niño decaying phase, RI occurrence positions migrate significantly westward toward the South China Sea and the Western Philippine Sea (SCS-WPS, 5–25°N, 110–140°E) (Fig. 1c) compared with neutral years (Supplementary Fig. 1). In contrast, the mean TC RI occurrence position, as well as the mean RI TC genesis positions for LD El Niño events undergo modest changes (Fig. 1d–f) compared with neutral years (Supplementary Fig. 1).

Quantitatively, 78.0% of TC RI occurs to the west of 140°E for the SD El Niño events, whereas only 40.0% of TC RI for LD El Niño events occur in that area. A previous study suggested that the La Niña events always last for 2–3 years^19^, which leads to a cooled central Pacific. Similarly, the central Pacific is also cooled during the SD El Niño decaying phase. However, there is no westward migration of RI occurrence position during La Niña events (Supplementary Fig. 2). This indicates that the central Pacific thermal state may have little effect on the westward migration of RI occurrence positions during SD El Niño events. The impacts of El Niño Modoki^20^ and its three subtypes^21^ on RI occurrence positions was also explored. No westward migration was observed during their decaying phases (Supplementary Fig. 3). Additionally, there is also no westward migration of RI occurrence positions during the SD and LD El Niño developing phases (JASON during the developing year) (Supplementary Fig. 4). In summary, the noticeable westward migration of RI occurrence position is observed only during the SD El Niño decaying phase. As the TC intensity has biases among different best-track datasets^22–24^, we employed two additional datasets, Japan Meteorological Agency (JMA) and China Meteorological Administration (CMA), to validate the Joint Typhoon Warning Center (JTWC). The composite RI occurrence position during the SD El Niño decaying phase for both JMA and CMA show obvious westward migration toward the SCS-WPS region (Supplementary Fig. 5). The numbers of RI TCs during the SD El Niño decaying phase show differences among the three datasets, which reflects some biases in TC intensity among these datasets. However, these biases do not undermine the results because three datasets consistently show westward migration of the RI occurrence positon during SD El Niño decaying phase. Additionally, the intensity change of the WNP TCs in JTWC shows better consistency with the large-scale environment conditions, such as the SST anomaly (SSTA) and the VWS than those of JMA and CMA^25^. This further enhances the reliability of the westward migration of the RI occurrence position during SD El Niño decaying phases.

To quantitatively evaluate the influence of SD El Niño events on the westward migration of the mean RI occurrence position, the composite difference of several RI-related variables between different types of El Niño events and the neutral years are shown in Supplementary Table 1. The composite difference in the mean RI occurrence longitudes between SD El Niño and neutral years is –8.04° (*P* = 0.001), but that for the mean TC genesis longitudes is only –5.56° (*P* = 0.108). This indicates the westward migration of TC RI occurrence position is not caused by the westward migration of RI TC genesis. The composite difference between the mean RI TC genesis longitude and the mean RI occurrence longitude is further compared for each of the three occasions: SD El Niño events, LD El Niño events, and neutral years. During SD El Niño events, the composite difference between mean RI TC genesis longitude and mean RI occurrence longitude is 7.98° (*P* = 0.0493), that in LD El Niño events is 4.88° (*P* = 0.1468), and that in neutral years is 5.5° (*P* = 2.89 × 10^–8^). These results further suggest that the westward migration of RI occurrence position during SD El Niño decaying phases is independent of the change in TC RI genesis position.

The composite difference in the mean RI occurrence time between SD El Niño events and the neutral years is 43.69 h (*P* = 0.023) over the eastern WNP. This indicates that the RI TCs formed over the open sea during SD El Niño events have a delayed RI occurrence relative to the neutral years (blue dots in Fig. 1e). As the TCs generally move westward with the tropical easterly wind, the delayed RI occurrence time must correspond to a westward migration of RI occurrence position. Interestingly, the composite difference of the mean RI occurrence time (red dots in Fig. 1e) between SD El Niño events and neutral years is –20.94 h (*P* = 0.005) over the western WNP. This means the RI TCs that formed over the western WNP during SD El Niño years occur earlier than that during neutral years on average. The above results imply that the environmental conditions over the western WNP during SD El Niño events may be more favorable for RI occurrence than that in the eastern WNP, which makes TCs formed remotely over open sea to move westward and begin RI. The mean RI duration time difference for SD El Niño events is 4.52 h (*P* = 0.078), which is longer than during neutral years. During LD El Niño events, all the above variables are not statistically significant. For the composite difference between all El Niño events and neutral years, only the RI duration time is statistically significant with 3.64 h (*P* = 0.024) longer than neutral years. This will not be further discussed because all other variables are not statistically significant.

The RI frequency and peak intensity are not statistically significant for both SD and LD El Niño events. For RI frequency, only four SD El Niño events and five LD El Niño events were involved in the composite analysis. The limited sample sizes may affect the statistical significance. Although the RI frequency difference is not statistically significant, we cannot rule out the possible impact of the LD and SD El Niño events on the RI frequency. The peak intensity is more complex than the position-related variables because it is generally decided by both the inner-core dynamics and the environmental conditions^26^. RI frequency may be closely related to the peak intensity^27^, but the composite difference in RI frequency and peak intensity are both statistically insignificant, and this may be the reason why their changes disagree with each other.

### The effects of atmospheric and oceanic factors

Three major climate factors, which include the VWS, RH, and TC heat potential (TCHP), may significantly affect the TC RI^7,28–30^. The VWS and RH are large-scale atmospheric dynamical and thermodynamic factors, respectively, and the TCHP is an oceanic factor. A strong VWS is generally detrimental to TC intensification^31–33^. Thus, on a variety of timescales, the VWS is generally anti-correlated with TC’s intensity change^34^. Conversely, a high RH in the mid-lower troposphere is favorable for TC intensification^33^. The third climate factor TCHP is widely used to represent the upper OHC and acts as a heat energy reservoir fueling the overlying atmosphere and TC intensification^7,13^. Hence, high TCHP favors TC RI. Generally, the TC RI tends to occur in the regions with warmer upper ocean thermal conditions, weaker VWS, and higher RH in the mid-lower troposphere. To investigate the mechanisms by which the two types of El Niño events lead to the zonal displacement of TC RI occurrence position, we analyzed the behaviors of these three climate factors during the SD and LD El Niño decaying phases.

**Fig. 2:**
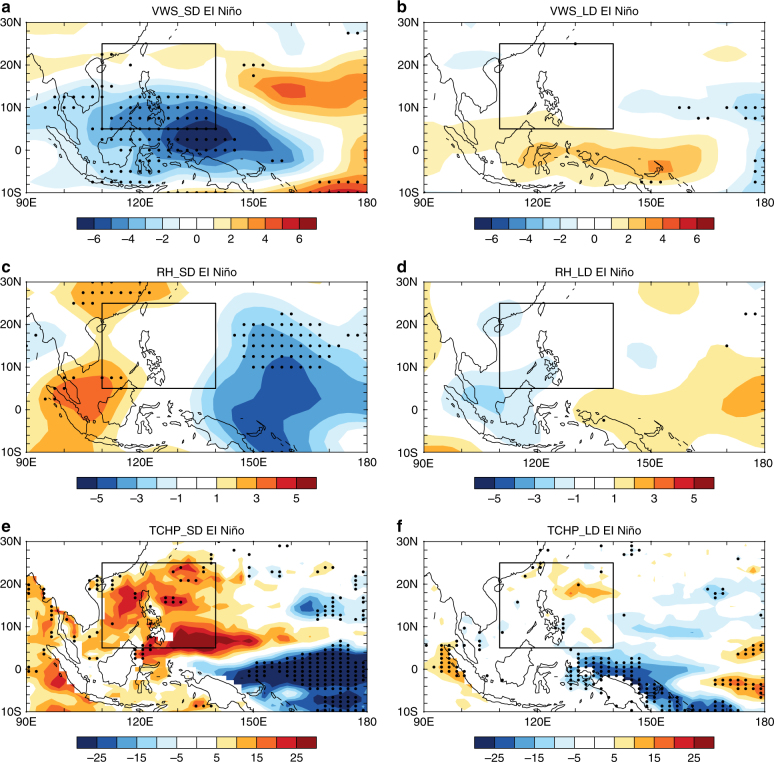
Large-scale climate factors associated with the short duration and long duration El Niño events. Composite anomalies of **a** vertical wind shear (units: m s^–1^), **c** relative humidity (units: %), and **e** tropical cyclone heat potential (units: 10^7^ J m^–2^) in July–November for the short duration El Niño events, **b**, **d**, and **f** as in **a**,** c**, and **e**, but for long duration El Niño events. Black boxes indicate the South China Sea and the Western Philippine Sea region. The vertical wind shear and relative humidity were from the NCEP-NCAR reanalysis dataset and the tropical cyclone heat potential was derived from the SODA reanalysis dataset. The stippled areas indicate the values are statistically significant at the 95% confidence level

Figure 2 shows the composite anomalies of the VWS, RH, and TCHP during SD and LD El Niño decaying phases. Large negative VWS anomalies are presented during SD El Niño decaying phases over the western Pacific warm pool region, which covers the southern part of the SCS-WPS region (Fig. 2a), whereas during LD El Niño decaying phases, there are only minor changes in VWS over the SCS-WPS region (Fig. 2b). The above evidence indicates that the VWS over the SCS-WPS region is much weaker during SD El Niño decaying phases than during LD El Niño decaying phases. This is further supported by a previous study that suggested that during strong El Niño decaying phases, the VWS over the SCS is weakened due to an eastward propagated warm Kelvin wave^35^. The RH shows positive anomalies over the SCS-WPS region during SD El Niño decaying phases (Fig. 2c), which can also be observed in the 40-year European Centre for Medium-Range Weather Forecasts Reanalysis (ERA-40) dataset (Supplementary Fig. 6). During LD El Niño decaying phases, RH has no obvious signals over the SCS-WPS region (Fig. 2d). Strong positive anomalies of TCHP are present during SD El Niño decaying phases over the SCS-WPS region (Fig. 2e), which is coincident with the region of concentrated RI occurrence during SD El Niño years (Fig. 1c). This indicates that the increase in TCHP over the SCS-WPS region during the SD El Niño decaying years plays an important role in shifting the RI occurrence positions to the west. In contrast, there is little change in TCHP over the SCS-WPS region during LD El Niño decaying phases (Fig. 2f). Thus, the mean RI occurrence position is close to that during neutral years (Fig. 1d). In summary, the weak VWS, moistened troposphere, and high TCHP over the SCS-WPS region are crucial to the westward migration of the RI occurrence position during SD El Niño decaying phases.

**Fig. 3:**
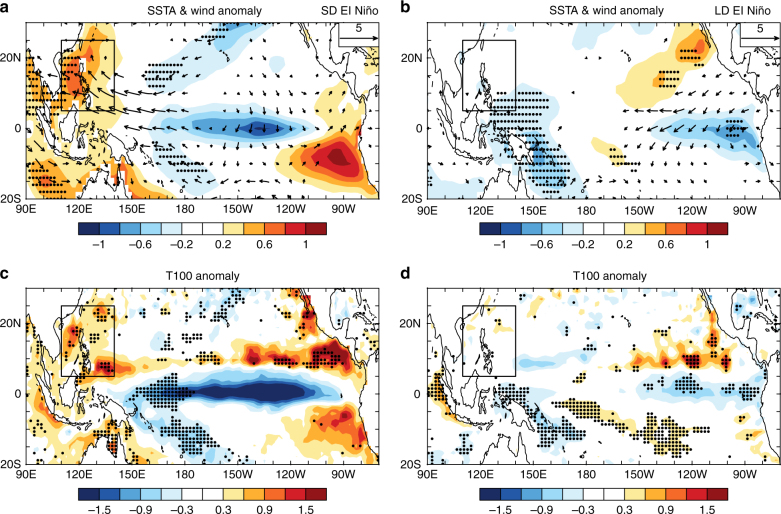
Low-level wind and upper ocean thermal conditions during the short duration and long duration El Niño events. Composite anomalies of **a** sea surface temperature (shading, units: K) from ERSST reanalysis dataset and 850-hPa wind (vectors, units: m s^–1^) from NCEP-NCAR reanalysis dataset, and **c** mean ocean temperature between 5 and 100 m (units: K) from SODA reanalysis dataset for the short duration El Niño events; **b**, **d** as in **a**,** c**, but for the long duration El Niño events. The stippled areas indicate the values are statistically significant at the 95% confidence level. Black boxes indicate the South China Sea and the Western Philippine Sea region. Wind anomalies <0.2 m s^–1^ are not shown

However, variations in VWS, RH, and TCHP do not occur independently of one another. The large-scale atmospheric circulation change, which leads to VWS anomalies, can result from the upper OHC-related SSTAs^35,36^, thus, the relationship among these factors deserves further discussion. Figure 3 shows the composite anomalies of the SST, 850-hPa horizontal wind, and T100 (defined as the ocean temperature anomalies averaged between 5–100 m) during SD and LD El Niño decaying phases. The SSTA can modify the large-scale circulation, as well as VWS, and the T100 anomaly may potentially indicate the change in the upper OHC and TCHP^12,37–39^. During SD El Niño decaying phases, the SSTA pattern is consistent with the anomalous TCHP pattern and is associated with noticeable warming over the SCS-WPS region (Fig. 3a). This increase in SST over the SCS-WPS region further increases the middle and lower troposphere moisture and induces a large zonal SSTA gradient over the WNP. The anomalous zonal SSTA gradient is located in the low latitudes, which is where the wind is balanced between surface pressure gradient force and the surface frictional force^40^. Therefore, the low-level wind anomalies flow along the downward gradient direction of the SSTA and correspond to the strong easterly wind anomalies (Fig. 3a, also see Supplementary Fig. 7). As the westerly wind prevails in the lower troposphere during JASON over the SCS-WPS region (Supplementary Fig. 8), the anomalous easterly wind during SD El Niño decaying phases can reduce the mean westerly wind, which further reduces the VWS over the SCS-WPS region. Additionally, the low latitudes feature thermal direct circulation, thus the low- and high-level winds are closely coupled. The composite wind anomalies at 200 hPa show a westerly (Supplementary Fig. 9), which is the opposite of the easterly presented at a low level, and thus, the westerly weakens the local VWS. Comparatively, both the SST and low-level horizontal wind show minor changes in LD El Niño events, and the VWS anomalies are not statistically significant over the SCS-WPS region (Fig. 3b). We also analyzed the VWS anomalies during the El Niño developing years, and found that they undergo either weak or insignificant changes over the SCS-WPS region (Supplementary Fig. 10), which induces no westward migration of RI TC genesis positions (Supplementary Fig. 4).

The VWS over the SCS-WPS region may also be weakened by other factors. It is suggested that Indian Ocean warming plays a role in affecting the VWS over SCS region during strong El Niño decaying phases^35,41^. During SD El Niño decaying phases, the northern Indian Ocean also shows warming anomalies (Supplementary Fig. 11a), which excites a warm baroclinic Kelvin wave propagating into the western Pacific, as indicated by the eastward stretch of the warm temperature anomalies, and thus, the baroclinic Kelvin wave leads to westerly VWS anomalies (Supplementary Fig. 11b). The climatological mean VWS over the western Pacific is easterly (Supplementary Fig. 11c), and thus, the mean easterly VWS is weakened by the westerly VWS anomalies induced by Indian Ocean warming. These results indicate that the Indian Ocean warming during SD El Niño decaying phases can also contribute to the negative VWS anomalies over the SCS-WPS region.

The T100 anomalies show similar patterns to those of the SST and TCHP over the SCS-WPS region (Fig. 3c, d). Additionally, the ocean temperature anomalies averaged over the SCS-WPS region undergo an overall warming above 200 m with a peak at ~80 m during SD El Niño decaying phases (Supplementary Fig. 12), whereas those which occur during LD El Niño decaying phases change little. This evidence indicates that the SST warming and TCHP increase over the SCS-WPS region during SD El Niño decaying phases can be attributed to the overall warming of the upper ocean.

There are other factors that may affect the RI change^42^. Although they may not be independent from the subsurface oceanic forcing, they delineate some other aspects of how SD El Niño events impact the TC RI. To begin with, the previous 12 h intensification rates are 4.08 per h (*P* = 0.4594) for SD El Niño events and –1.17 per h (*P* = 0.7607) for LD El Niño events, both of which are statistically insignificant, and this indicates that the previous intensity change may not affect the WNP TC RI change. Not all predictors can affect the RI within a single ocean basin. The dominant predictors are different among various ocean basins^42^. Over the SCS-WPS region during SD El Niño decaying phases, the 200-hPa divergence shows positive anomalies, and the vertical velocity anomalies indicate upward motion in the entire troposphere (Supplementary Fig. 13a, b). These factors may enhance the upper-level outflow and middle-level inflow, and strengthen the upward motion near the storm’s inner core^43^, which is favorable for the rapid deepening of a TC. The maximum potential intensity, which is strongly tied to the change in TC intensity and locations, shows positive anomalies over the SCS region (Supplementary Fig. 13c), and this indicates the potential intensification of TCs over this region. However, these three factors show nearly no signals over the SCS-WPS region during LD El Niño decaying phases (Supplementary Fig. 13d–f). These factors are closely related to the underlying SST change, which further highlights the important role of the oceanic dynamics in affecting the TC RI westward migration during SD El Niño decaying phases.

In summary, the westward migration of the RI occurrence position during SD El Niño decaying phases is closely related to the change in the oceanic conditions. The SD El Niño decaying phase leads to an increase in the upper OHC (indicated by T100) over the SCS-WPS region. The SSTA can be affected by both the upward oceanic heat transport and the surface heat flux. Similar to the OHC pattern, there are positive anomalies for the SSTA over the SCS-WPS region. This indicates that the upper OHC plays a role in transporting oceanic heat to the sea surface to offset the cooling effect caused by TC or other factors, which maintains the SSTA as positive. The SST warming over the SCS-WPS region combined with SST cooling over the middle Pacific can lead to a weakened VWS over the SCS-WPS region, which provides a favorable condition for TC genesis and intensification. However, the TC intensification needs persistent energy fueling. The positive TCHP over the SCS-WPS region indicates that the OHC increase is large enough to provide persistent energy fueling for the TC intensification. In brief, the SD El Niño induced OHC increase over the SCS-WPS region is large enough to maintain a positive SSTA and provide extra energy (indicated by positive TCHP anomalies) for fueling the TC intensification.

### Mechanisms for the upper ocean heat content change

**Fig. 4:**
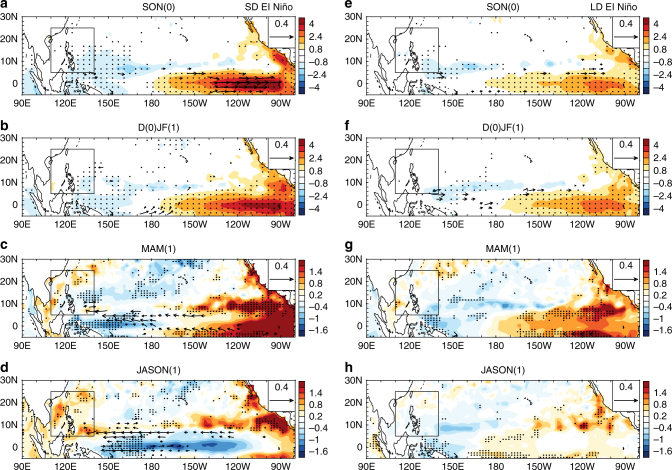
Evolution of the ocean heat content and upper ocean current for the short duration and long duration El Niño events. Composite anomalies of mean upper ocean (5–100 m) temperature (shading, units: K) and currents (vectors, units: m s^–1^) from SODA reanalysis dataset for **a** September(0)–November(0), **b** December(0)–February(1), **c** March(1)–May(1), and **d** July(1)–November(1) during short duration El Niño events. **e**–**h** as in **a**–**d**, but for long duration El Niño events. (0) and (1) indicate the months in El Niño developing and decaying years, respectively. Black boxes indicate the South China Sea and the Western Philippine Sea region. The stippled areas indicate the values are statistically significant at the 95% confidence level. The vectors only show the currents anomalies exceeding the 95% confidence level

A question arises as to how the SD and LD El Niño events lead to the different anomalous upper OHC patterns over the SCS-WPS region. To answer this question, changes in the T100 during both types of El Niño decaying phases were explored. From December(0) to February(1), both the SD and LD El Niño events reach their peaks and coincide with subsurface warming in the middle and eastern Pacific, and subsurface cooling in the western Pacific (Supplementary Fig. 14), which resemble the SSTA patterns in the mature phases of all El Niño events. The middle and eastern Pacific subsurface temperature during SD El Niño decaying phases are warmer than during LD El Niño decaying phases. However, during JASON(1), the composite T100 anomalies of the SD and LD El Niño events are different (Fig. 3c, d). The SD El Niño events show significant cooling of the equatorial subsurface and warming of the off-equatorial subsurface (around 10°N) over the tropical Pacific, along with warming of the SCS-WPS subsurface (Fig. 3c), which is consistent with those of the SST and TCHP anomalies (Figs. 3a, 2e). In contrast, there is no subsurface warming over the SCS-WPS region during LD El Niño decaying phases, as indicated by the T100 anomalies, and the subsurface remains warm around 180° south of the equator during LD El Niño decaying phases (Fig. 3d). During both SD and LD El Niño decaying phases, the eastern Pacific (east of 150°W) off-equatorial subsurface is warm (Fig. 3c, d). The recharge–discharge oscillator provides a physical explanation for the subsurface temperature change in the eastern Pacific^18^. The cooled equatorial subsurface and warmed off-equatorial subsurface are caused by a meridional discharge of the OHC driven by Sverdrup transport (Supplementary Fig. 15). Then, the eastern North Pacific subsurface warming leads to TC intensification over the warmed region^12^. The OHC increase over the SCS-WPS region during SD El Niño decaying phases may also be caused by discharging effects, but a zonal advection of OHC is necessary for this process. To validate this hypothesis, Fig. 4 shows the evolution of the T100 and the subsurface current anomalies during SD and LD El Niño decaying phases, respectively. During the SD El Niño decaying phase, a strong westward zonal current is established at ~10°N (Fig. 4a–d), which coincides with the latitude of the off-equatorial subsurface warming. The zonal currents converge to the western boundary of the Pacific basin leading to the SCS-WPS subsurface warming (Fig. 3c). The zonal currents burden a westward advection of the discharged OHC during the El Niño decaying phases. Zonal currents also appear during the LD El Niño decaying phases, but they only extend as far as the middle Pacific (Fig. 4e–h). Thus, the SCS-WPS is not warmed, but the Niño-3.4 region subsurface continues to be warm throughout the LD El Niño event, which leads to a prolonged decaying phase.

The westward currents first appear at the eastern and western boundaries of the Pacific basin in the SD El Niño developing year, and then they propagate to the middle Pacific and reverse the original eastward currents over the middle Pacific around December(0) (Supplementary Fig. 16). This process plays an important role in terminating El Niño events after they reach their peak phases, which is followed by a rapid decaying (Fig. 1a). Comparatively, the westward currents during the LD El Niño decaying phases evolve in a similar manner to those during SD El Niño decaying phases, but the westward currents are relatively weak, and the middle Pacific eastward currents persist throughout the lifespan of an El Niño (Supplementary Fig. 16), which prolongs the decaying phases (Fig. 1b). These observations are consistent with an advective–reflective oscillator^16^: the central Pacific westerly produces upwelling Rossby (downwelling Kelvin) waves propagating westwards (eastwards) which are reflected as upwelling Kelvin (downwelling Rossby) waves after reaching the western (eastern) boundaries. The upwelling Kelvin wave and downwelling Rossby wave can produce westward zonal currents, which is consistent with the observed the boundary-originated zonal currents (Supplementary Fig. 16). The westerly anomalies that occur during SD El Niño decaying phases are much larger than those during LD El Niño events (Supplementary Fig. 17), which may lead to stronger upwelling Kelvin wave and downwelling Rossby wave during the SD El Niño decaying phases as well as stronger zonal currents (Fig. 4).

## Discussion

By analyzing the observational datasets, we found that the SD El Niño events led to the westward migration of the WNP TC RI occurrence position, with the mean RI occurrence position much closer to the East Asian mainland. The zonal advection of the upper OHC from the eastern Pacific to the SCS-WPS region plays an important role in the westward migration of the TC RI occurrence positions during SD El Niño events. The RI occurrence positions migrate east during both the SD and LD El Niño developing phases (Supplementary Fig. 4). This may be caused by the eastward advection of OHC (Fig. 4a, e), which is opposite to that of the SD El Niño decaying phases. This further suggests the important role of the OHC transport in affecting the TC RI occurrence positions. A previous study emphasized the role of atmospheric processes associated with tropical Pacific SSTA in affecting the WNP TC intensity^8^, while this study suggested that the oceanic heat transport from the eastern Pacific to the SCS-WPS region has crucial impacts on the zonal displacement of RI occurrence positions. These results have important implications for the seasonal prediction of the RI occurrence positons over WNP during El Niño decaying years.

The insignificant relationship between LD El Niño and the RI occurrence positions may be due to not enough ocean heat being transporting from the eastern Pacific to the SCS-WPS region. However, the underlying mechanism involves the oceanic dynamics during El Niño events. One possible reason is that the westerly wind during LD El Niño development phase is much weaker than that of the SD El Niño events (Supplementary Fig. 17), which may lead to a relatively shallower thermocline during LD El Niño’s mature phase. The SSTA peak phases of SD El Niño events are larger than LD El Niño events, and the different depths of the thermocline indicates large differences in the recharged energy going into the eastern Pacific, which may lead to different oceanic and atmospheric conditions during their decaying periods.

Strong El Niño events tend to be SD El Niño events^44^ and strong El Niño frequency is projected to increase under global warming^45^. Combined with the results in this study, there may be more near-land TC RI occurrence because of high-emission scenarios, which will narrow the time window for disaster prevention and mitigation from strong TCs and aggravate the TC destructiveness in the future. In addition, the TCs that make landfall over the WNP have been intensified during the past three decades because of ocean surface warming on the rim of East and Southeast Asia^46^. We demonstrate that over the WNP not only the SSTA, but also an overall warming of the upper ocean over the SCS-WPS region, are responsible for the westward migration of TC RI occurrence positions, which highlights the important role of upper ocean thermal dynamics in affecting the near-land TC intensification over the WNP. Therefore, these results may provide a new perspective for understanding the WNP TC intensity changes, as well as their future projection.

## Methods

### Datasets

The best-track TC dataset used in this study was from the JTWC, which covers the period 1950–2010^47^. The TC dataset has six-hourly information for individual TCs, which include the maximum sustained wind speed, minimum pressure, and geographical location. We also employed the JMA and CMA best-track datasets^48^ to validate the JTWC. Because of the uncertainties in the TC intensity estimation, the intensity error in the current best-track datasets can reach 10 kt over the Atlantic Ocean^22–24^. Over the WNP, the JTWC best-track is suggested to have an intensity error of nearly 6 kt^46^. By incorporating the new satellites and aircraft reconnaissance, the intensity error may be decreased in some extent, but this leads to temporal heterogeneity in the datasets^22^. Given the intensity error and the temporal heterogeneity in the best-track datasets, our results may contain uncertainties. Therefore, it is still necessary to search for more observational evidence to further enhance the reliability of the current results. The monthly mean zonal wind for the period 1950–2010 was derived from the National Centers for Environmental Prediction-National Center for Atmospheric Research (NCEP-NCAR) reanalysis, which has a horizontal resolution of 2.5° × 2.5° in longitude and latitude^49^. The 40-year European Centre for Medium-Range Weather Forecasts Reanalysis (ERA-40)^50^ was employed to validate the RH anomalies in the NCEP-NCAR reanalysis. The SST dataset used to analyze the evolution of El Niño events was from the Extended Reconstructed SST version 3b (ERSST), which has a horizontal resolution of 2.0° × 2.0° in longitude and latitude^51^. The monthly mean oceanic temperature, and zonal and meridional currents were obtained from the Simple Ocean Data Assimilation (SODA) dataset, which has a horizontal resolution of 0.5° × 0.5° in longitude and latitude and 40 vertical layers^52^.

### El Niño events

The El Niño events were identified using the 3-month running mean Niño-3.4 indices (area-averaged SSTA over the region of 5°S–5°N, 120–170°W) >0.5 °C. The monthly Niño-3.4 index has shown an upward trend since the 1950s. To avoid the influence on the identification of the El Niño events, the monthly Niño-3.4 indices of each 5-year periods are calculated using different 30-year base periods to remove the warming trend of Niño-3.4 index following the Climate Prediction Center (CPC). For instance, the Niño-3.4 indices during 1950–1955 will be based on the 1936–1965 base period, Niño-3.4 indices during 1956–1960 will be based on the 1941–1970 base period, and so on and so forth. The trend has influence on El Niño magnitude, but does not influence the classification of SD and LD El Niño events (Supplementary Fig. 18). Using the above criterion and detrended Niño-3.4 index, nine El Niño events (1951/1952, 1957/1958, 1965/1966, 1972/1973, 1976/1977, 1982/1983, 1986/1987, 1991/1992, and 1997/1998) and nine La Niña events (1955/1956, 1967/1968, 1970/1971, 1973/1974, 1975/1976, 1984/1985, 1988/1989, 1999/2000, and 2005/2006) were identified. An SD El Niño is defined as having a Niño-3.4 index that becomes negative and is <–0.5 °C during the decaying summer and autumn; an LD El Niño is defined as having a Niño-3.4 index that generally stays >0 °C throughout the period of decay^14^. Thus, among the nine El Niño events, the SD El Niño events are 1972/1973, 1982/1983, 1986/1987, and 1997/1998, and the LD El Niño events are 1951/1952, 1957/1958, 1965/1966, 1976/1977, and 1991/1992. El Niño Modoki events were categorized into three subtypes according to their evolutionary patterns^21^: prolonged decaying (1968/1969, 1990/1991, and 1991/1992), the abrupt decaying (1963/1964, 1977/1978, and 1987/1988), and the symmetric decaying (1994/1995, 2002/2003, and 2004/2005). For all El Niño events, the first year is the developing year and the second year is the decaying year. The months in developing years are denoted by (0), while the months in decaying years are denoted by (1). When excluding the SD El Niño, LD El Niño, El Niño Modoki, and La Niña events, the remaining years during 1950–2010 are defined as neutral years. As the El Niño’s evolution has phase-locking feature with mature phase in boreal winter, we choose 2 years’ time windows to evaluate the SD and LD El Niño events, with the first year denoting the developing phase and the second year denoting the decaying phase. The decaying phase of LD El Niño events can last longer than 1 year, but we still use 1 year as the decaying phases of both SD and LD El Niño events because the two types of El Niño events can already be distinguished in the first year during their decaying phase, and the focus of this study is also in the first decaying year.

### Calculation of oceanic variables

The TCHP represents the OHC contained in the water warmer than 26 °C. It is calculated as follows^53^:

1 $${\mathrm{TCHP}} = {{\rm c}}_{\rm p}\rho {\int}_{D_{26}}^0 {\left[T\left( z \right) - 26\right]{{\rm d}}z},$$

where *c*_p_ is the heat capacity of the seawater at a constant pressure, which is taken as 4178 J kg^–1^ °C^–1^; *ρ* is the density of the seawater, which is taken as 1026 kg m^–3^ in the upper ocean; *D*_26_ is the depth of the 26 °C isotherm; and *T*(*z*) is the in situ temperature. T100 is the vertically averaged ocean temperature between the depth of 5–100 m, while the SST is the temperature at the ocean surface. The T100 can represent the upper OHC, while the TCHP indicates the portion of OHC that can fuel the TC activity. Thus, T100 changes can affect both the SST and TCHP anomalies. In addition, the SST changes can be affected by other factors, such as the solar radiation and cloud, and it is also important in driving the large-scale circulation. To estimate an approximate contribution of T100 to TCHP and SST, the SCS-WPS regional mean T100 anomalies are correlated with those of the TCHP and SST. It turns out that the correlation coefficient is 0.94 (88% explained variance) between the T100 and TCHP anomalies and is 0.78 (61% explained variance) between the T100 anomalies and SSTA. T100, TCHP, and SST are dynamically connected and their physical mechanism is unique, that is, the more time the TCs are moving over the ocean, the warmer subsurface water needed (a larger T100 is needed to provide TCHP) because TCs can cool the surface water (SST) and induce upwelling.

### Tropical cyclone rapid intensification-related definitions

The time a TC reaches the storm intensity (maximum surface sustained wind ≥17 m s^–1^) is defined as the genesis. A TC RI is defined as having a maximum sustained wind speed increase of at least 30 kt (15.4 m s^–1^) within a 24-h period^33^. As the definitions of RI vary^54,55^, to verify the robustness of the results, we checked the results based on the definitions of 35 kt per 24 h, 40 kt per 24 h, 45 kt per 24 h, and 15 kt per 12 h, respectively. The westward migration of the RI occurrence position can be observed for the other choices of RI definitions.

The RI occurrence position indicates the geographic position where a TC begins to undergo an RI. The RI occurrence longitude indicate the longitude of the RI occurrence positon. The RI TC genesis position indicates the genesis position of a TC that experienced at least one RI in its life. The RI TC genesis longitude indicate the longitude of the RI TC genesis position. The RI occurrence time indicates the hours a TC takes to begin a RI since its genesis, that is, the length of time between a TC genesis and its RI occurrence. The RI duration time is the temporal length of the first RI process. RI frequency indicates the total number of RI TCs during JASON. Peak intensity indicates the lifetime maximum intensity of a RI TC.

### Composite analysis and the statistical significance

The composite anomalies of the variables throughout this paper is based on the difference between El Niño’s decaying years and the neutral years, unless stated otherwise. As the autocorrelation may reduce the denominator of degrees of freedom, the *t* test with effective degrees of freedom (EDF) was imposed to the composite anomalies of RI-related variables, and the atmospheric and oceanic climate factors. To calculate the EDF, we first calculate the decorrelation timescale of the series involved in the composite analysis. For the RI-related variables, the autocorrelation is applied to the series composed by the RI TCs, and because they are not autocorrelated (Supplementary Fig. 19), their EDFs can be represented by the sample sizes. It is possible that the insignificant autocorrelations can slightly reduce the EDFs for some variables. However, such influence may be within a limited extent and do not qualitatively affect the results. For the atmospheric and oceanic variables, we first define the indices of these variables by averaging their anomalies over the SCS-WPS region, and then apply the autocorrelation to these indices. As the atmospheric and oceanic factors during SD (time series of *N* = 4 samples) and LD (*N* = 5) El Niño years are discrete annual values, they are also not autocorrelated. We still calculated their autocorrelations with 1-year lag and the results turn out to be insignificant, thus the EDFs are equal to their sample sizes. During neutral years (*N* = 44), the VWS, TCHP and T100 are not autocorrelated, while RH and SST have 2-year decorrelation timescales (Supplementary Fig. 20). Although there may exist biases in the decorrelation timescales of RH and SST and the biases also reduce half of their degrees of freedom, the composite differences of these variables are still statistically significant at the 95% confidence level over the SCS-WPS region (Figs. 2 and 3). Because the oceanic variables such as SST and TCHP may persist for several months, their decorrelation timescales may be <1 year. We further calculated the decorrelation timescales of the time series constituted by the monthly values of those involved in the composite analysis during SD El Niño, LD El Niño, and neutral years (Supplementary Fig. 21). The longest decorrelation timescales are 14 months for TCHP during neutral years. Supplementary Table 2 provides the decorrelation timescales, sample sizes, and EDFs of these variables. The composite differences of these variables based on their monthly values are consistent with those based on annual values and have even higher confidence levels (Supplementary Figs 22 and 23).

### Maximum potential intensity

The maximum potential intensity is an important estimate of the upper boundary of a TC’s intensity^56^. The maximum potential intensity calculation is based on the code provided by K.A. Emanuel (Massachusetts Institute of Technology) on his website. It can be calculated as follows:

2 $$V_{\mathrm{m}}^2 = \frac{{T_{\mathrm{s}}}}{{T_0}}\frac{{C_{\mathrm{k}}}}{{C_{\mathrm{D}}}}\left[ {{\mathrm{CAPE}} ^\ast - {\mathrm{CAPE}}} \right],$$

where *V*_m_ is the maximum gradient wind speed, *T*_s_ is the SST, *T*_o_ is the mean outflow temperature, *C*_k_ is the exchange coefficient for enthalpy, *C*_D_ is the drag coefficient, CAPE* is the convective available potential energy of air lifted from saturation at sea level, which is in reference to the environmental sounding, and CAPE is that of boundary layer air. We use the monthly mean specific humidity, sea level pressure, and air temperature from NCEP-NCAR, and the SST from the ERSST to calculate the maximum potential intensity.

### Data availability

The original observational datasets used in this study are publicly available and can be downloaded from the respective websites (JTWC, JMA, CMA, ERSST, NCEP-NCAR, ERA40, SODA).

## Electronic supplementary material

Supplementary Information
