# Supplementary material for: Westward migration of tropical cyclone rapid-intensification over the Northwestern Pacific during short duration El Niño
Source: Nat Commun. 2018 Apr 17;9:1507. doi: 10.1038/s41467-018-03945-y (PMC5904124; doi:10.1038/s41467-018-03945-y)
Supplement: Supplementary file 1 — Supplementary Information [file 41467_2018_3945_MOESM1_ESM.pdf]

Westward Migration of Tropical Cyclone Rapid-Intensification over the Northwestern Pacific during Short Duration El Niño

Yi-Peng Guo and Zhe-Min Tan*

Key Laboratory of Mesoscale Severe Weather/Ministry of Education, and School of Atmospheric Sciences, Nanjing University, Nanjing, China

Nature Communications

*Corresponding author: Zhe-Min Tan, E-mail: zmtan@nju.edu.cn

Contents of this file: Supplementary Figures 1–23 and Tables 1–2.

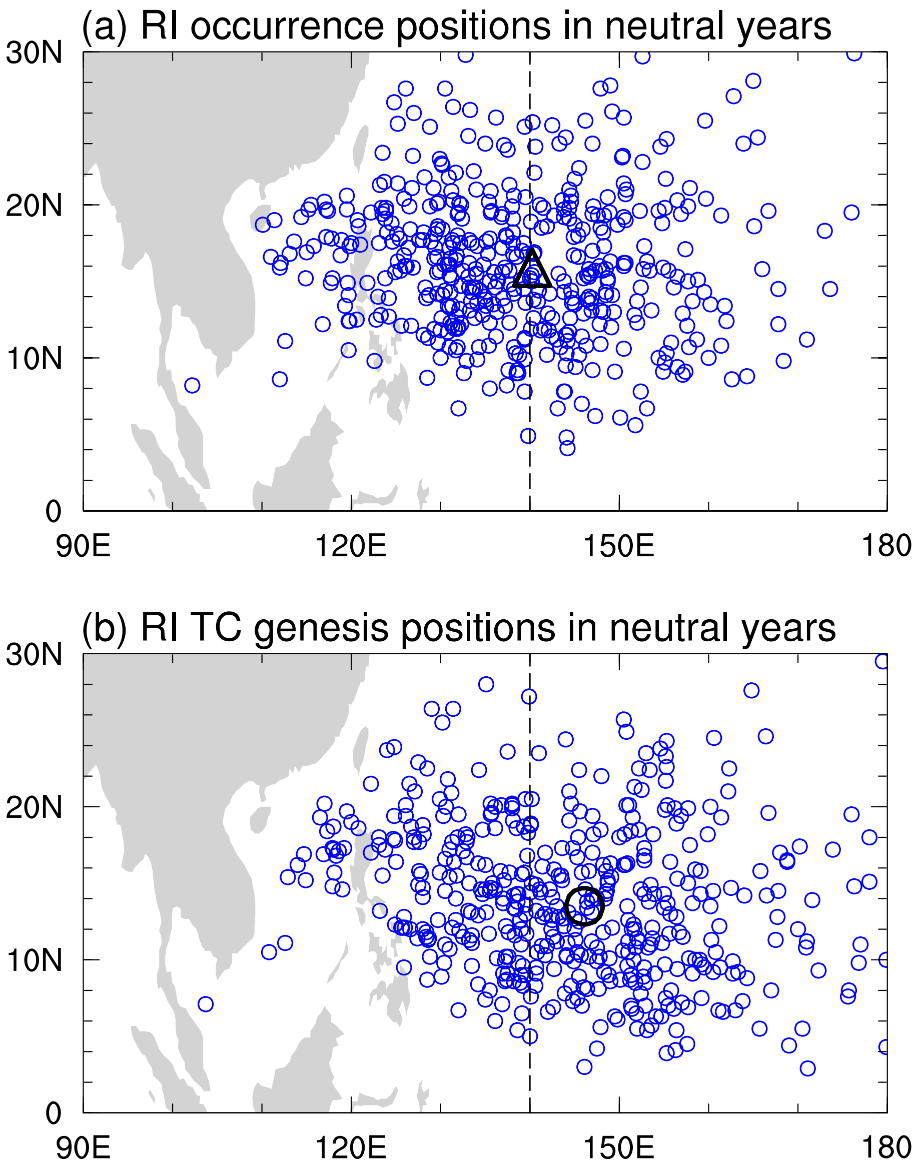

Supplementary Figure 1. Rapid intensification occurrence positions and rapid intensification tropical cyclone genesis positions during neutral years The composites of (a) rapid intensification (RI) occurrence positions and (b) RI tropical cyclone (TC) genesis positions for neutral years. The triangle and circle indicate the mean RI occurrence position and mean RI TC genesis position in the neutral years, respectively. The dashed lines indicate the 140°E longitude.

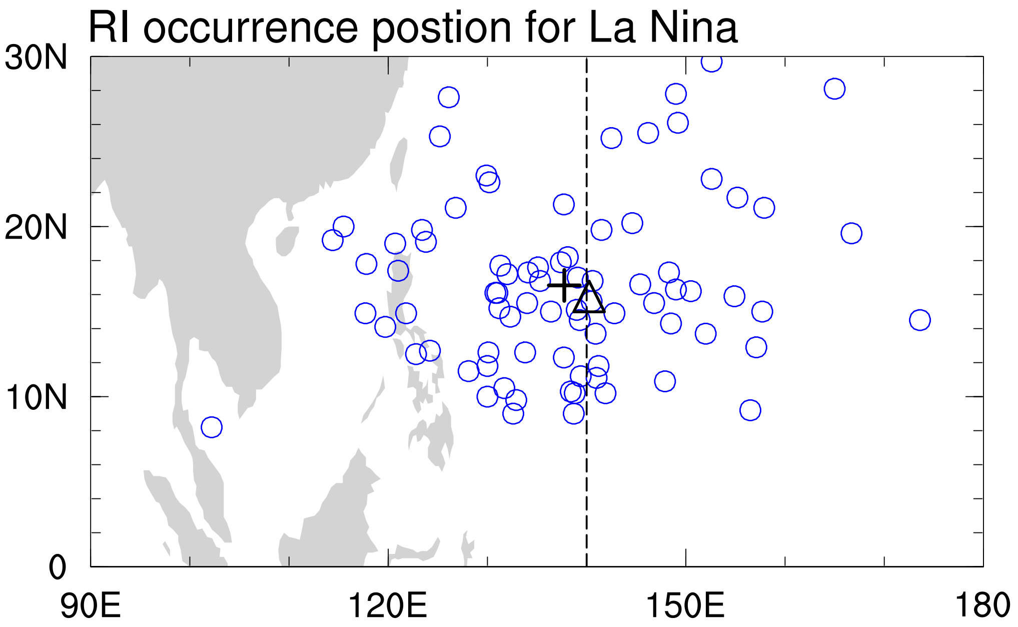

Supplementary Figure 2. Rapid intensification occurrence positions during La Niña years The composites of rapid intensification (RI) occurrence positions for La Niña decaying years. The cross and triangle indicate the mean RI occurrence positions for La Niña events and neutral years, respectively. The dashed lines indicate the 140°E longitude.

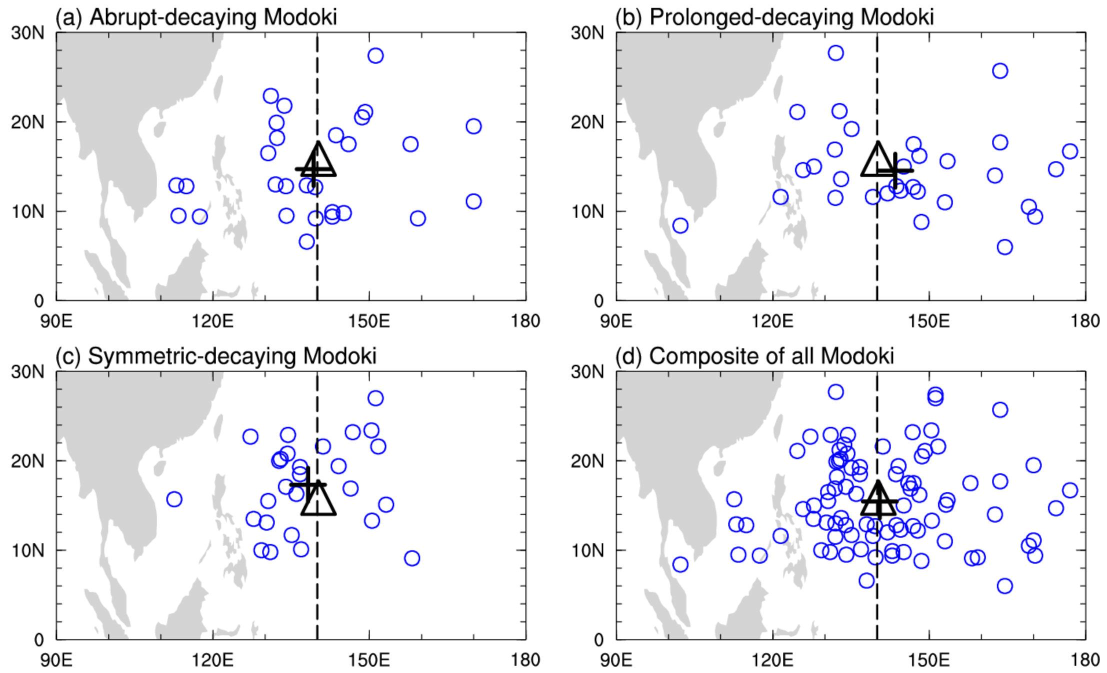

Supplementary Figure 3. Rapid intensification occurrence positions during El Niño Modoki years The composite rapid intensification (RI) occurrence positions for (a) abrupt-decaying Modoki events, (b) prolonged-decaying Modoki events, (c) symmetric-decaying Modoki events and (d) all Modoki events. The triangles in (a–d) indicate the mean RI occurrence position for neutral years, and the crosses indicate mean RI occurrence positions of corresponding types of Modoki events, respectively. The dashed lines indicate the 140°E longitude.

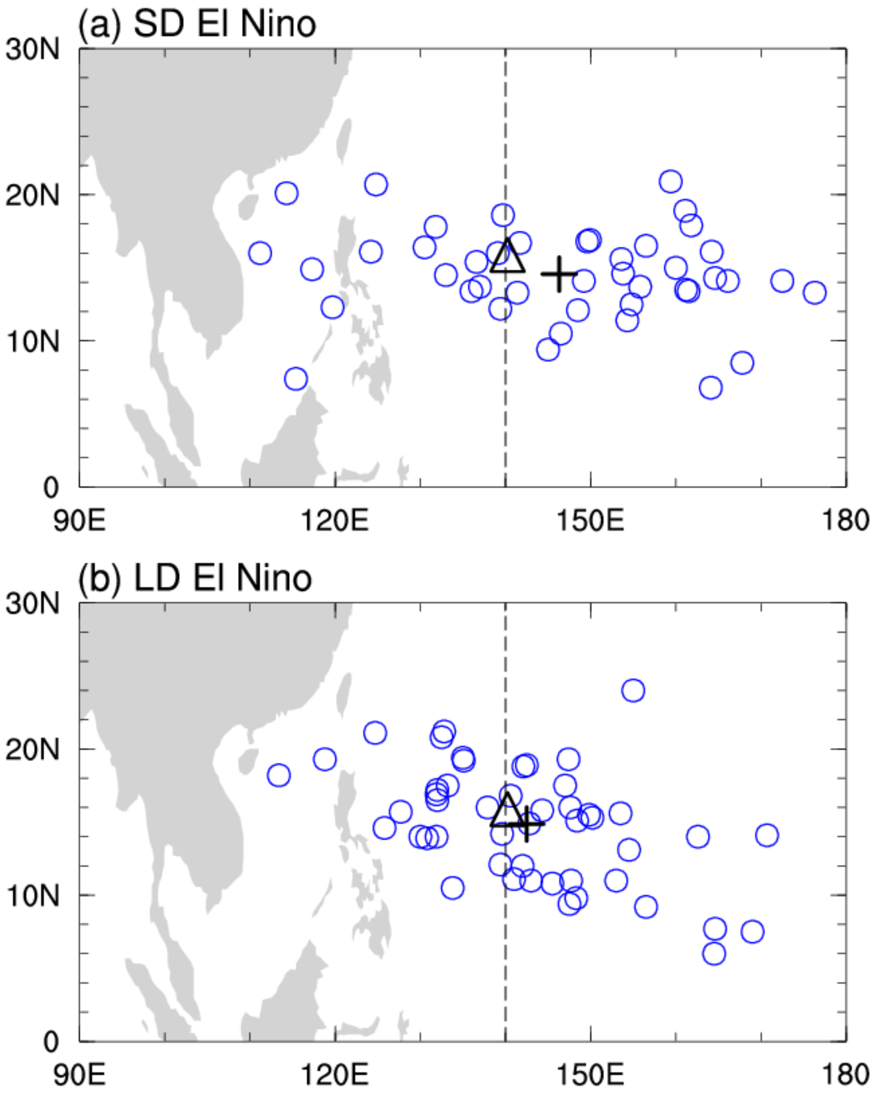

Supplementary Figure 4. Rapid intensification occurrence positions during El Niño developing years The composite rapid intensification (RI) occurrence positions for (a) short duration (SD) El Niño developing years and (b) long duration (LD) El Niño developing years. The crosses indicate the mean RI occurrence positions for (a) SD El Niño developing years and (b) LD El Niño developing years, respectively. The triangles indicate the mean RI occurrence position for neutral years. The dashed lines indicate the 140°E longitude.

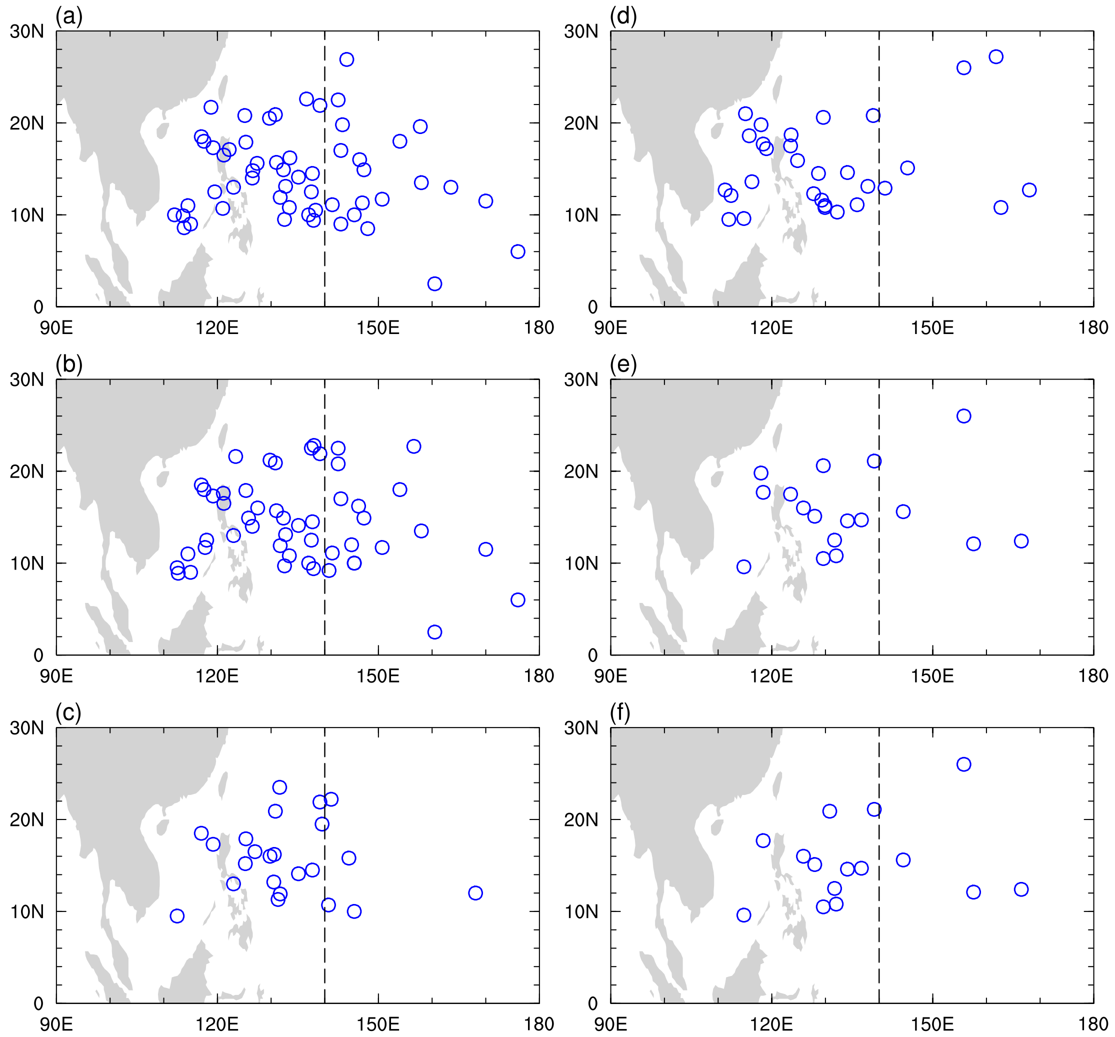

Supplementary Figure 5. Westward migration of rapid intensification occurrence positions in different tropical cyclone best-track datasets Composite rapid intensification (RI) occurrence positions for short duration El Niño years based on JMA (left panels) and CMA (right panels) best track datasets. The criteria to define RI for JMA are (a) 30 kt per 24 hrs, (b) 40 kt per 24 hrs and (c) 50 kt per 24 hrs, and for CMA are (d) 30 kt per 24 hrs, (e) 35 kt per 25 hrs and (f) 40 kt per 24 hrs. The dashed lines indicate the 140°E longitude.

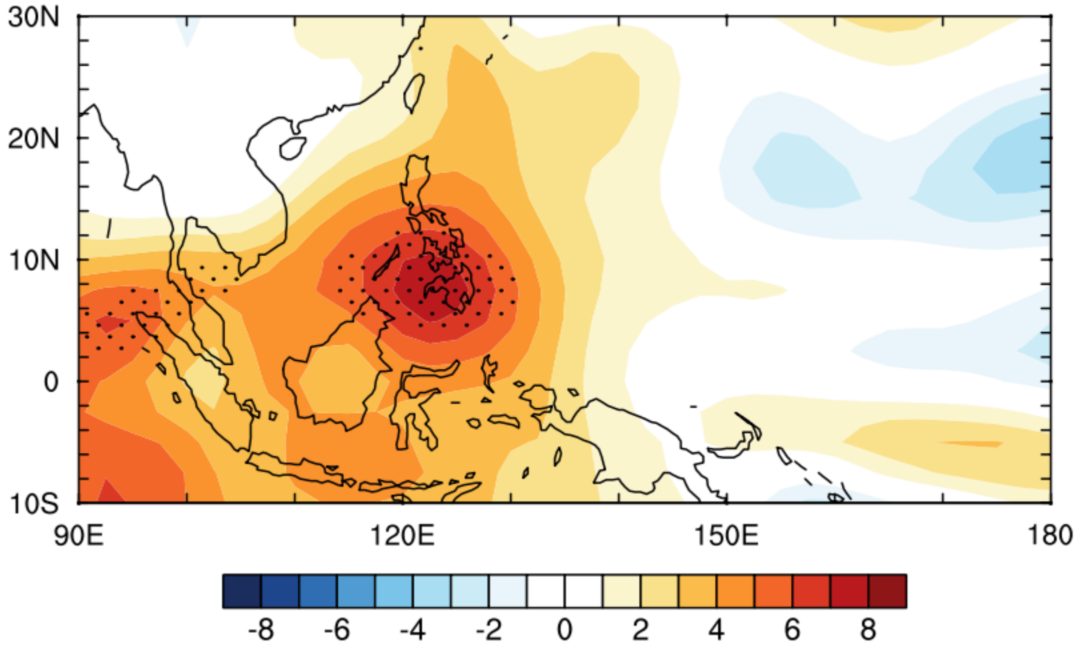

Supplementary Figure 6. Moistening of troposphere in short duration El Niño events Composite relative humidity anomalies for short duration El Niño decaying years. The stippled areas indicate the values exceeding the 95% confidence level.

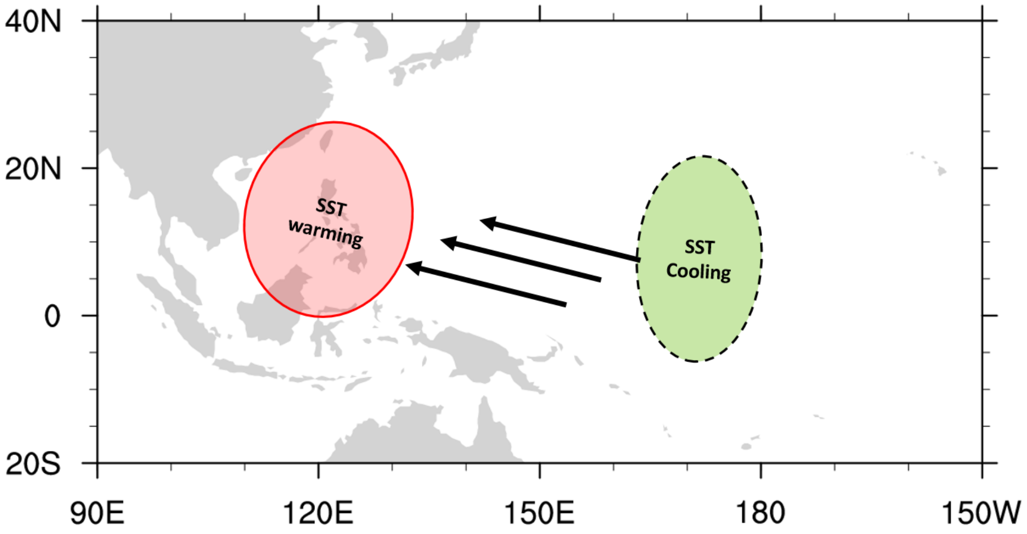

Supplementary Figure 7. Schematic for the relationship between sea surface temperature gradient and surface wind over the western Pacific Due to the warming of the South China Sea and the western Philippine Sea region (denoted by red ellipse) and cooling of the middle Pacific (denoted by green ellipse), the zonal sea surface temperature (SST) gradient over the western North Pacific (WNP) is enlarged. The enlarged zonal SST gradient leads to anomalous easterly wind (denoted by black arrows) over the WNP.

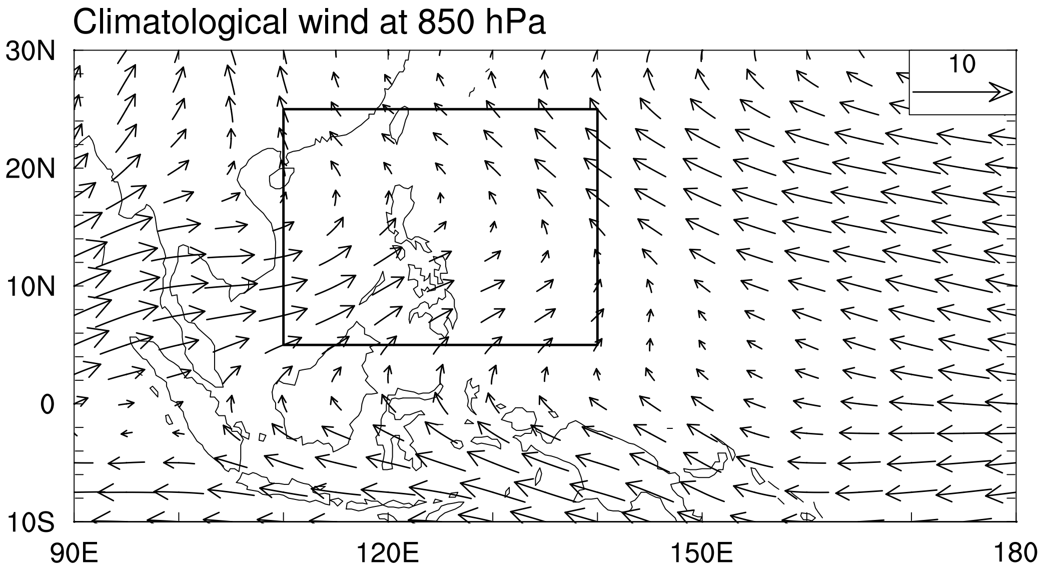

Supplementary Figure 8. Low-level wind climatology Climatological wind at 850 hPa in July–November based on the period 1950–2010. The black box indicates the South China Sea and the western Philippine Sea region.

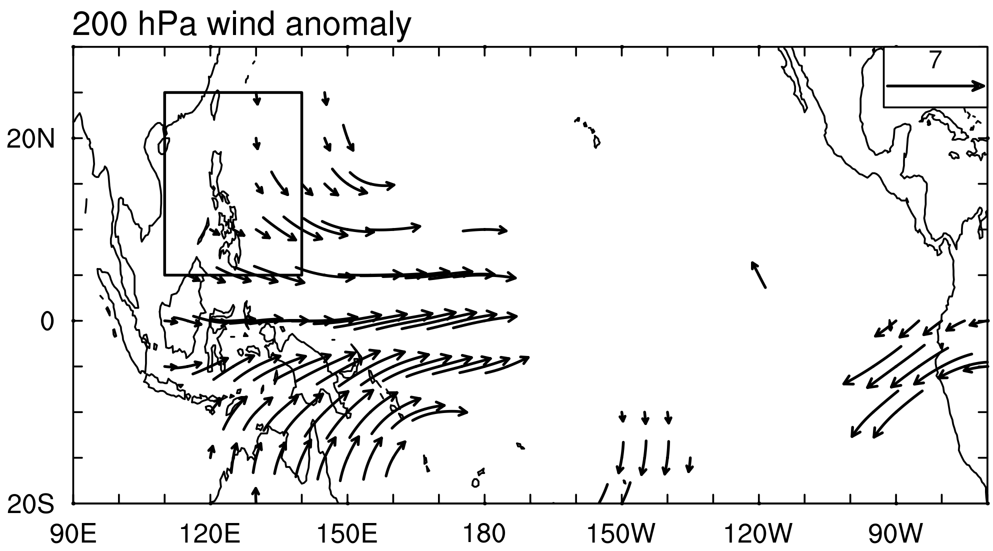

Supplementary Figure 9. 200 hPa wind anomalies Composite anomalies of the 200 hPa horizontal wind for short duration El Niño years. The wind vectors only show the anomalies exceeding the 95% confidence levels. The black box indicates the South China Sea and the western Philippine Sea region.

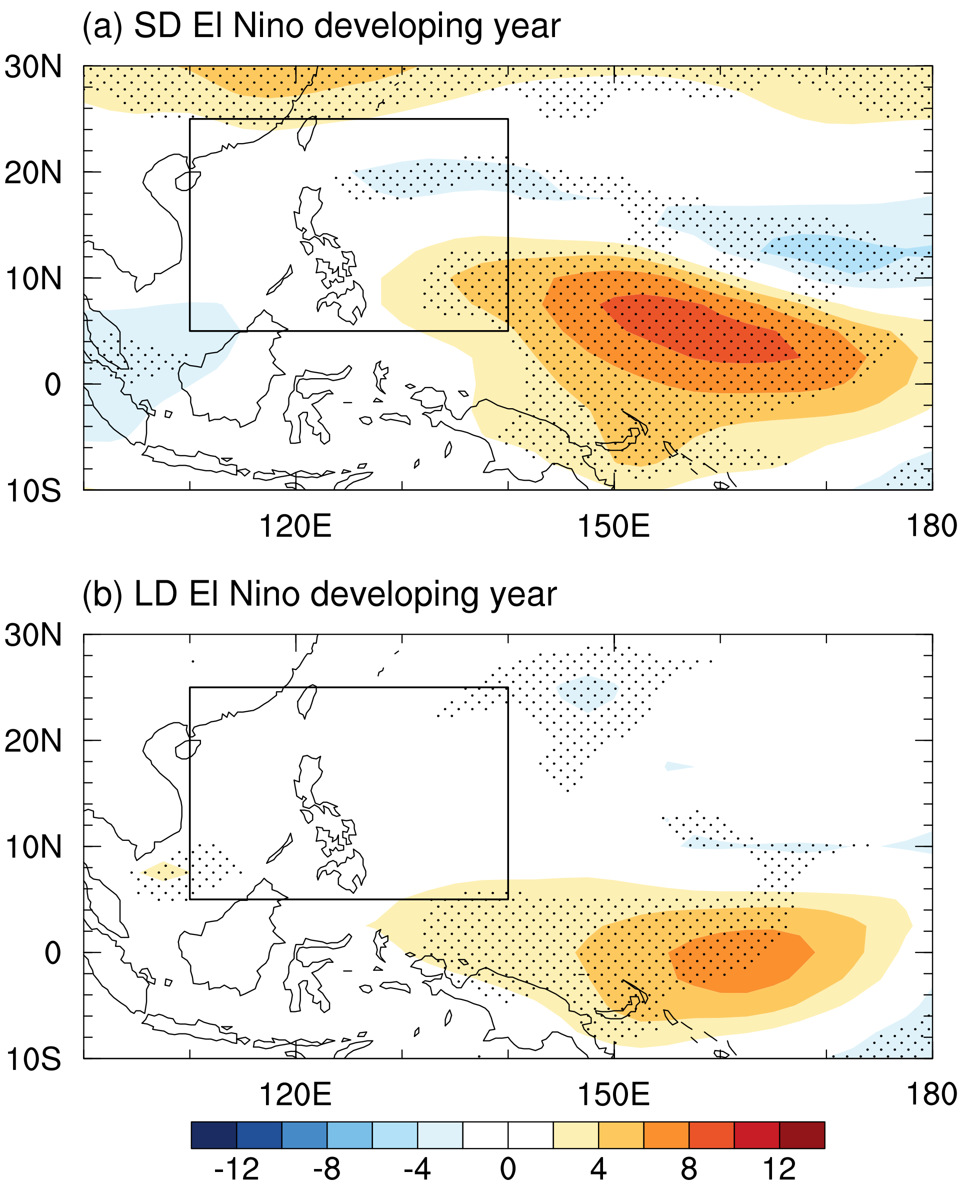

Supplementary Figure 10. Vertical wind shear anomalies in short duration and long duration El Niño developing years Composite vertical wind shear anomalies (shading, units: m s^{-1}) between 200 hPa and 850 hPa for (a) short duration El Niño developing years and (b) long duration El Niño developing years. The stippled areas indicate the values are statistically significant at the 95% confidence level. The black boxes indicate the South China Sea and the western Philippine Sea region.

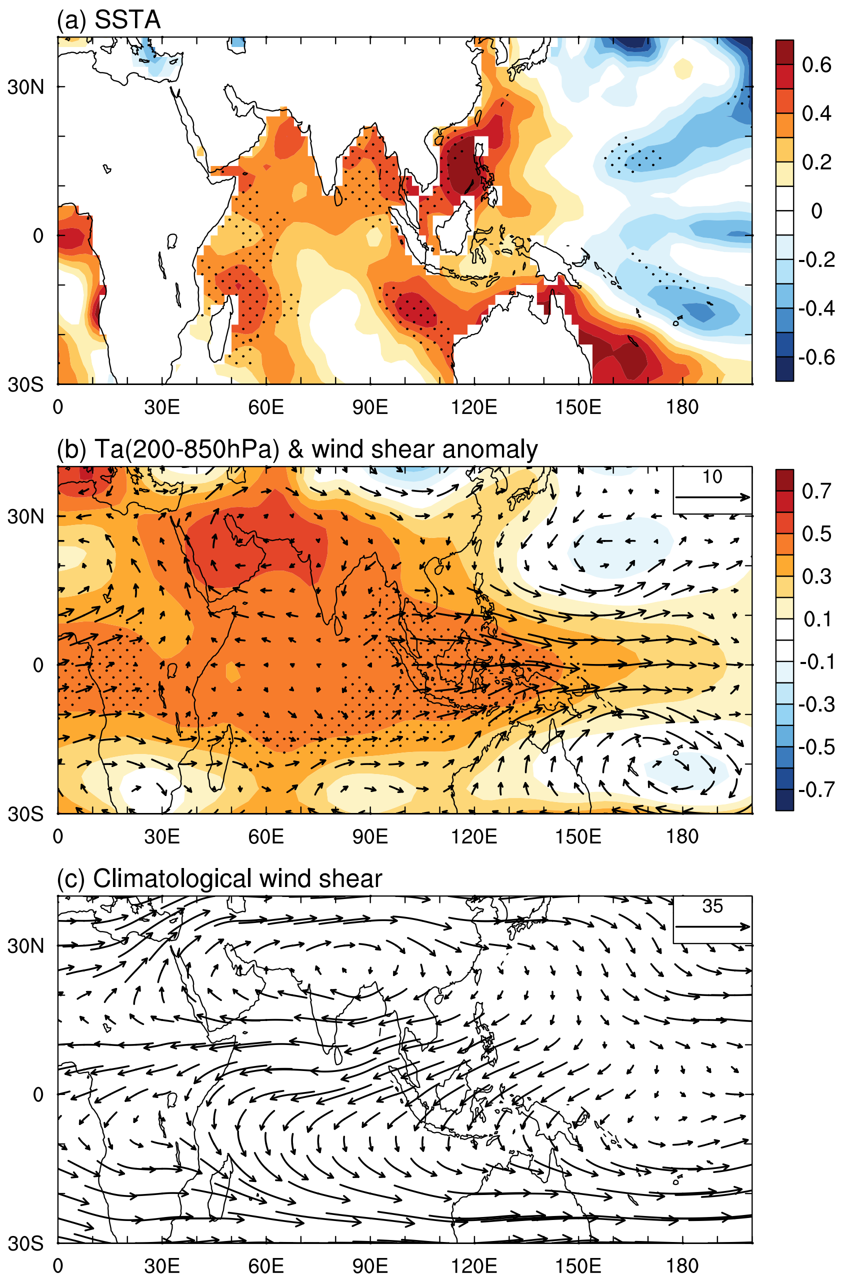

Supplementary Figure 11. Impacts from Indian Ocean Composite anomalies of (a) sea surface temperature (units: K) and (b) air temperature averaged between 200 hPa and 850 hPa (shading, units: K) and vertical shear of horizontal wind between 200 hPa and 850 hPa (vectors, units: m s^{-1}) for the short duration El Niño events. (c) shows the climatological mean vertical shear of the horizontal wind between 200 hPa and 850 hPa based on the period 1950–2010. The stippled areas indicate the values are statistically significant at the 95% confidence level.

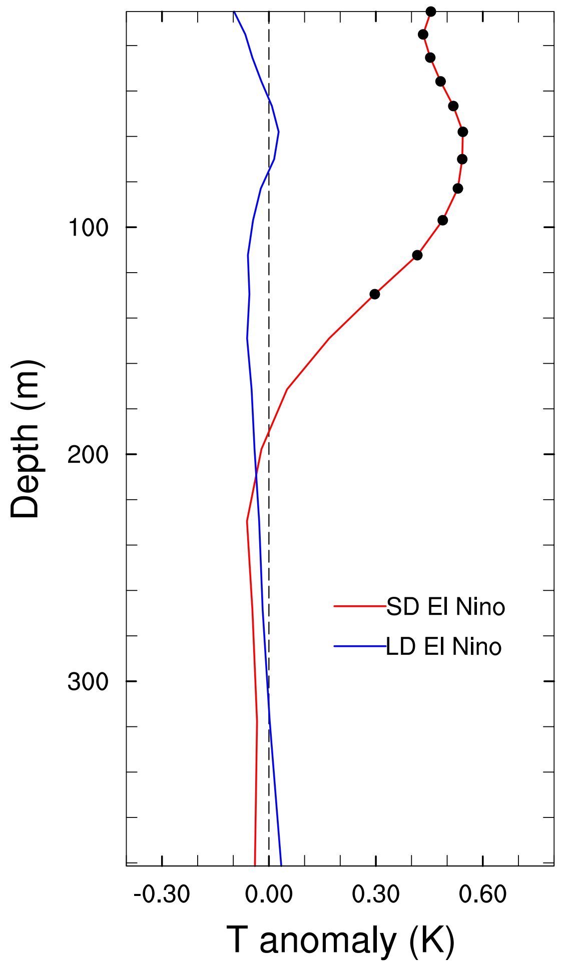

Supplementary Figure 12. Ocean temperature profiles Composite vertical profile of ocean temperature anomalies (units: K) over the South China Sea and the western Philippine Sea region for short duration El Niño years (red line) and long duration El Niño years (blue line), respectively. The black dots on the line indicate the values are statistically significant at the 95% confidence level.

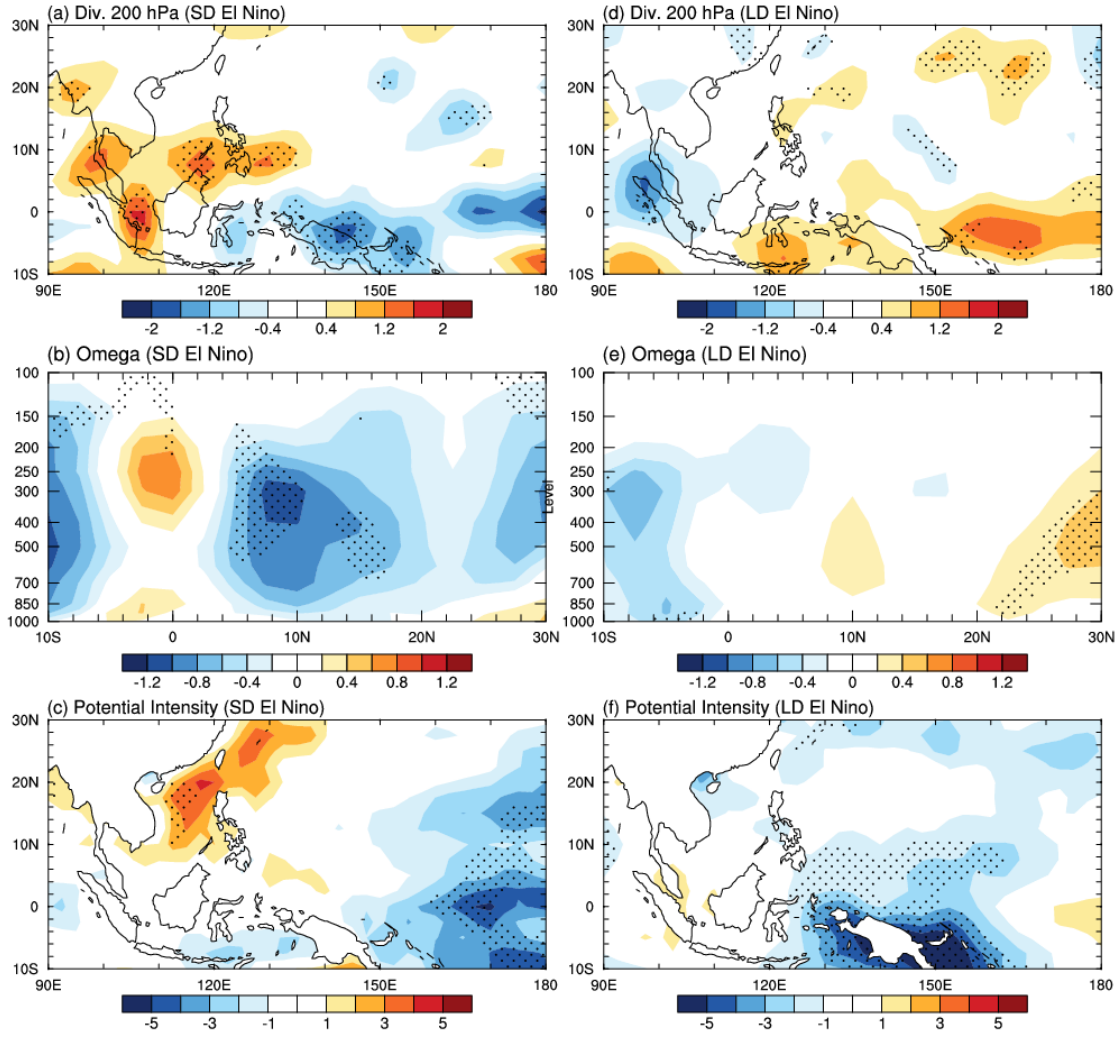

Supplementary Figure 13. Anomalies of environment factors Composite anomalies of (a) divergence at 200 hPa (10^{-6} s^{-1}), (b) vertical motion (10^{-2} Pascal s^{-1}) and (c) maximum potential intensity (m s^{-1}) for short duration El Niño events. (d–f) As in (a–c), but for long duration El Niño events. The stippled areas indicate the values are statistically significant at the 95% confidence level.

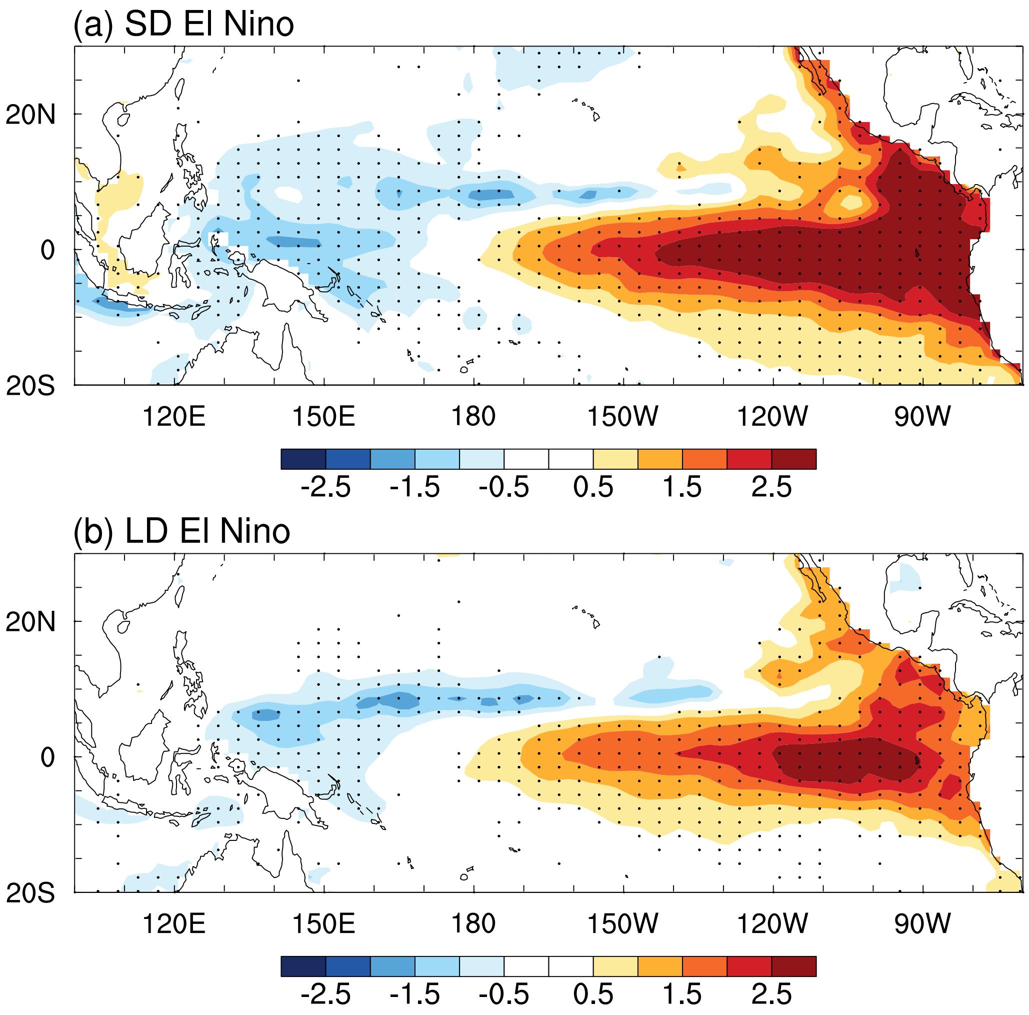

Supplementary Figure 14. Ocean heat content anomalies Composite anomalies of December–February mean ocean temperature between 5–100 m (K) for (a) short duration El Niño years and (b) long duration El Niño years. The stippled areas indicate the values are statistically significant at the 95% confidence level.

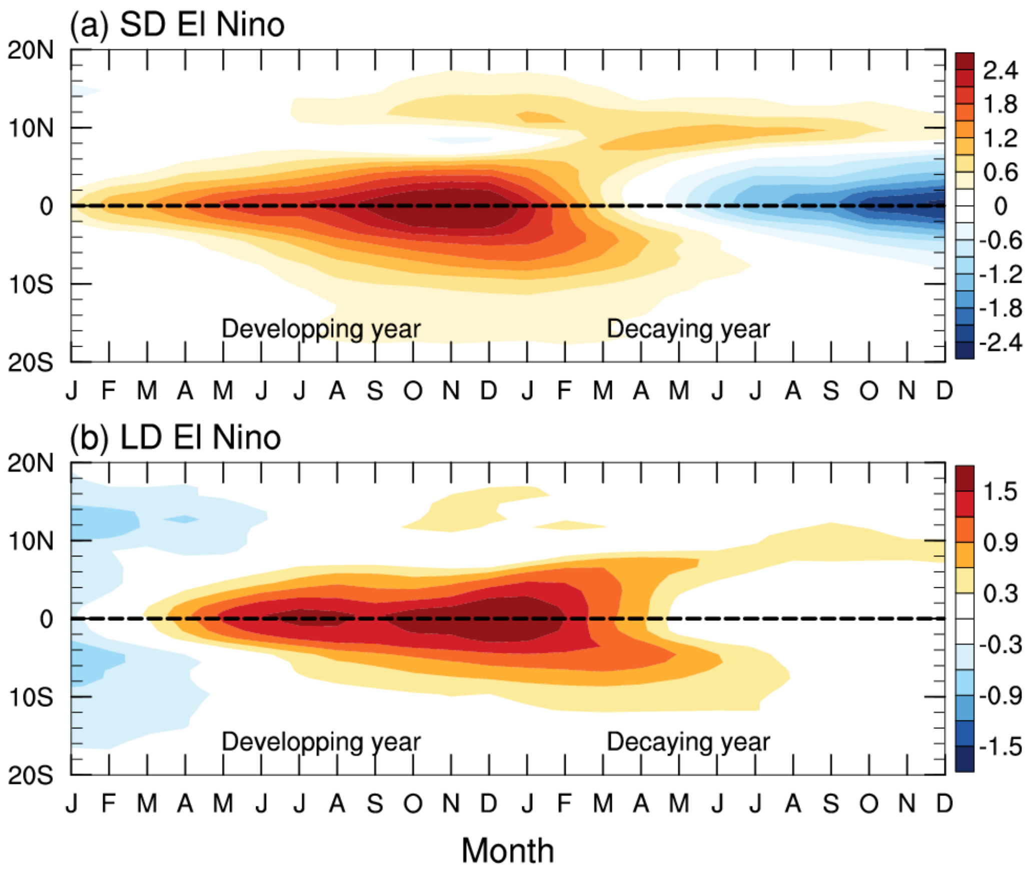

Supplementary Figure 15. Latitude-time cross section of the ocean heat content anomalies Composite evolutions of mean ocean temperature anomalies between 5–100 m (K) averaged over the middle and eastern Pacific (180°–70°W) for (a) short duration El Niño and (b) long duration El Niño events.

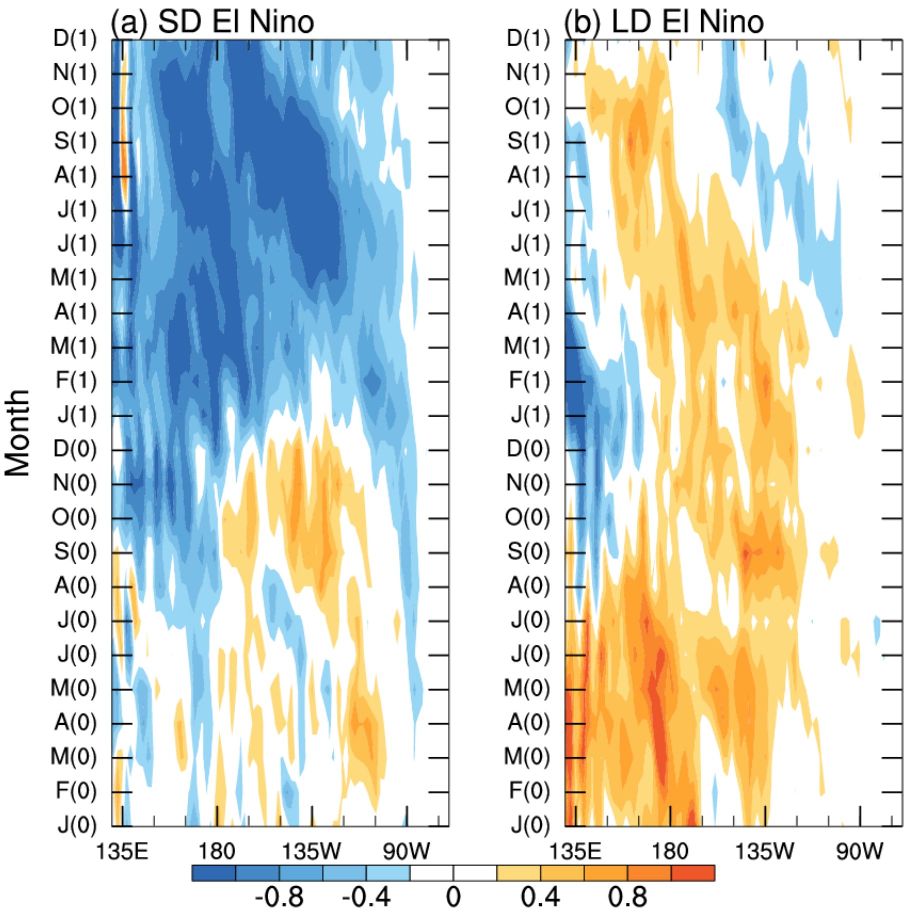

Supplementary Figure 16. Longitude-time cross section of the ocean zonal current anomalies Composite evolutions of the upper ocean (5–100 m) zonal current anomalies averaged over the Northern Hemisphere equatorial region (5°–10°N) for (a) short duration El Niño and (b) long duration El Niño events, respectively. The vaules are multiplied by a factor of 10.

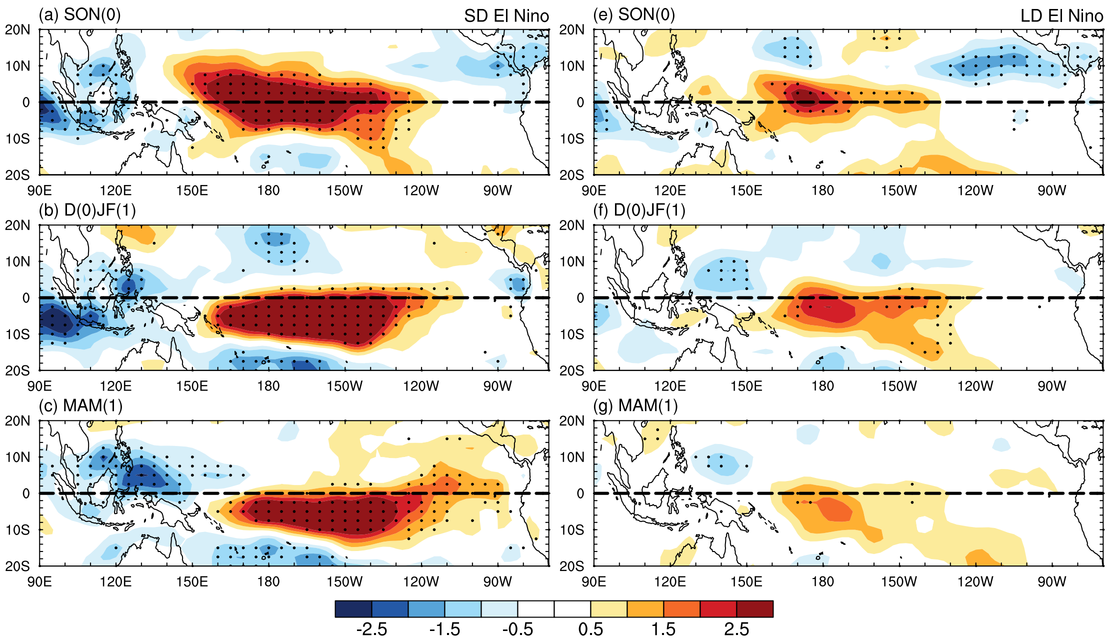

Supplementary Figure 17. Evolution of zonal winds anomalies Composite 1000 hPa zonal wind anomalies (units: m s^{-1}) in (a) September(0)–November(0), (b) December(0)–February(1) and (c) March(1)–May(1) for short duration El Niño events. (e–g) as in (a–c), but for long duration El Niño events. The stippled areas indicate the values are statistically significant at the 95% confidence level.

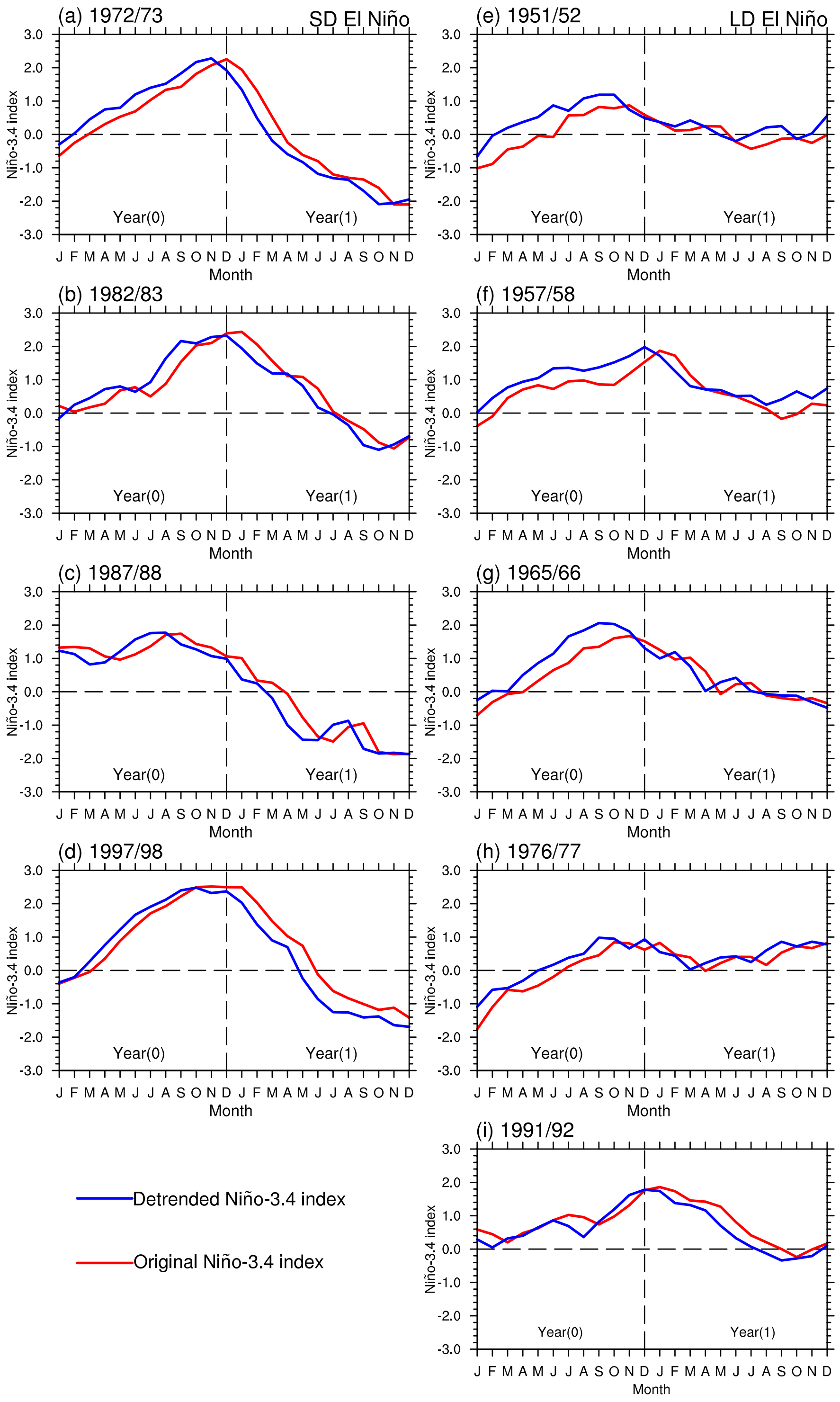

Supplementary Figure 18. Evolution of El Niño events Evolution of the original (red lines) and the detrended (blue lines) Niño-3.4 indices for short duration El Niño events (left panels) and long duration El Niño events (right panels).

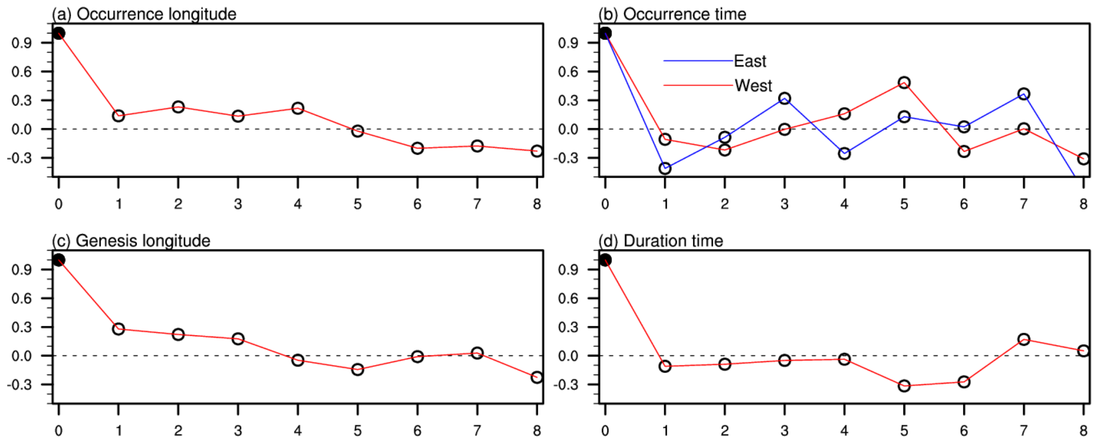

Supplementary Figure 19. Auto-correlation of rapid intensification related variables Auto-correlation of mean rapid intensification (RI) (a) occurrence longitude, (b) occurrence time, (c) genesis longitude, and (d) duration time. The filled black circles denote significant correlation at a confidence level of 95%. Blue and red lines in (b) indicate the RI tropical cyclones formed in the east and west of 140°E, respectively.

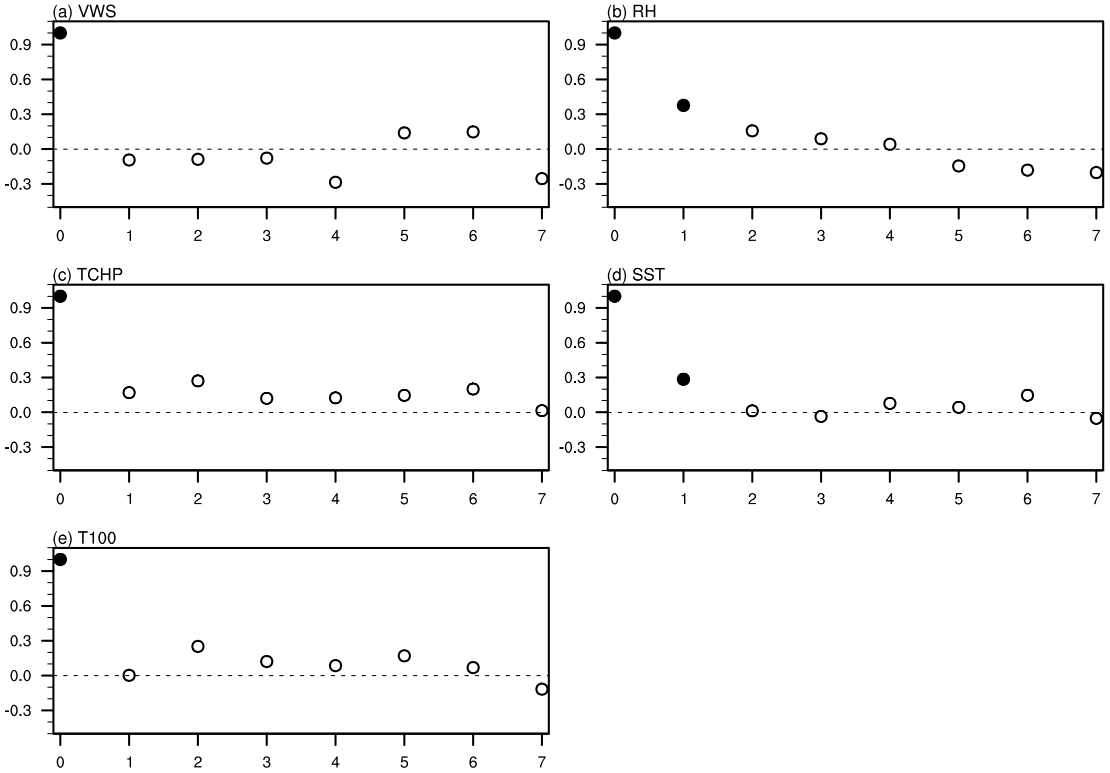

Supplementary Figure 20. Auto-correlation of atmospheric and oceanic variables Auto-correlation of indices of (a) vertical wind shear, (b) relative humidity, (c) tropical cyclone heat potential, (d) sea surface temperature and (e) averaged ocean temperature between 5–100 m of neutral years. The filled black circles denote significant correlation at a confidence level of 95%.

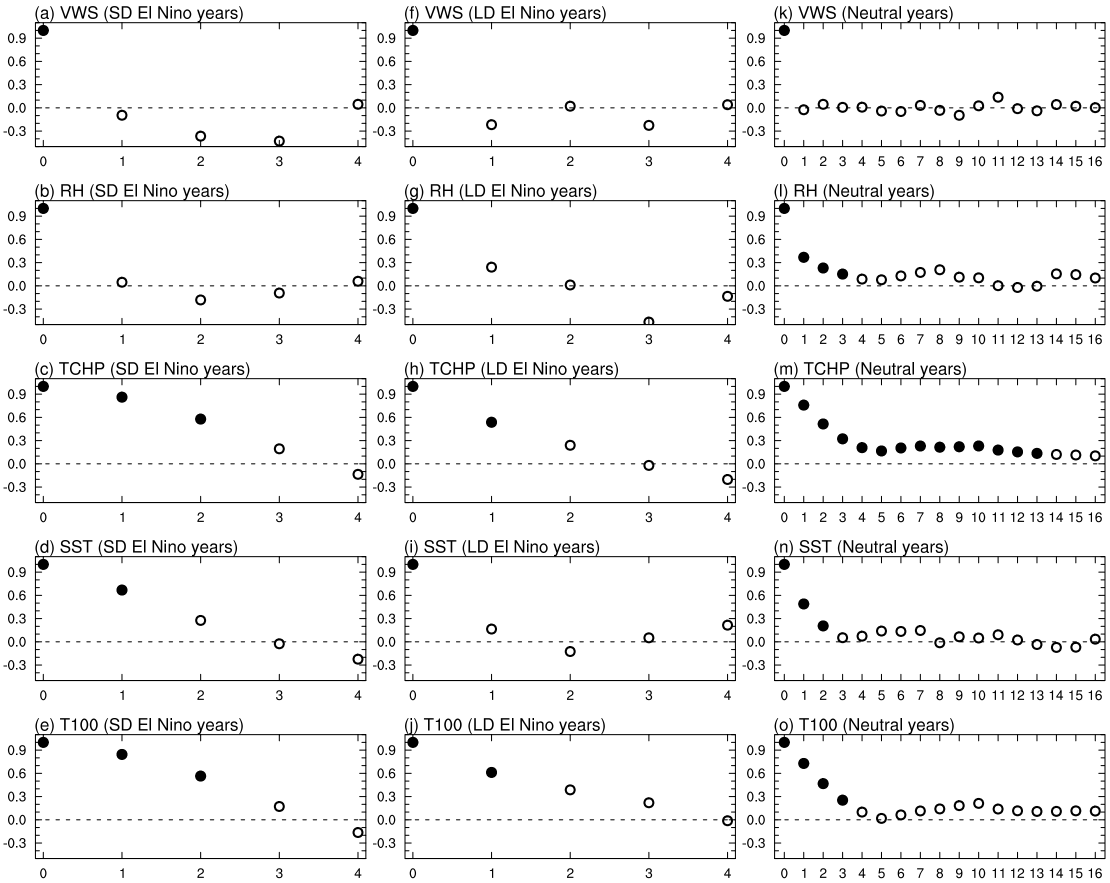

Supplementary Figure 21. Monthly auto-correlation of atmospheric and oceanic variables Auto-correlation of indices of (a) vertical wind shear, (b) relative humidity, (c) tropical cyclone heat potential, (d) sea surface temperature and (e) averaged ocean temperature between 5–100 m of short duration El Niño years. (f–j) and (k–o) are same as (a–e), but for long duration El Niño and neutral years, respectively. The filled black circles denote significant correlation at a confidence level of 95%.

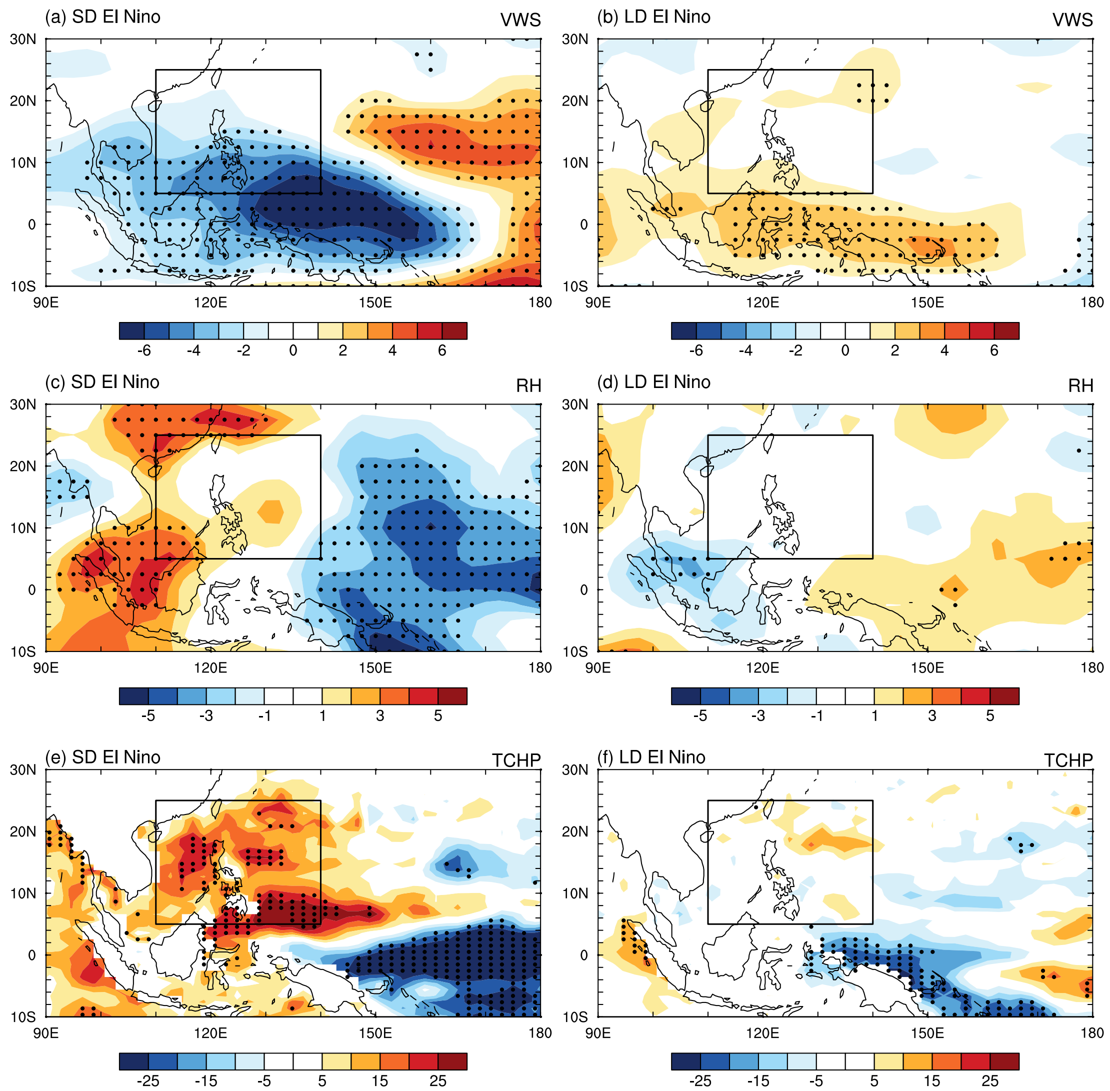

Supplementary Figure 22. Large-scale climate factors associated with the short duration and long duration El Niño events Composite anomalies of monthly (a) vertical wind shear (units: m s^{-1}), (c) relative humidity (units: %), and (e) tropical cyclone heat potential (units: 10^7 J m^{-2}) in July–November in the short duration El Niño decaying years, (b, d and f) as in (a, c, and e), but for long duration El Niño events. Black boxes indicate the South China Sea and the western Philippine Sea region. The stippled areas indicate the values are statistically significant at the 95% confidence levels.

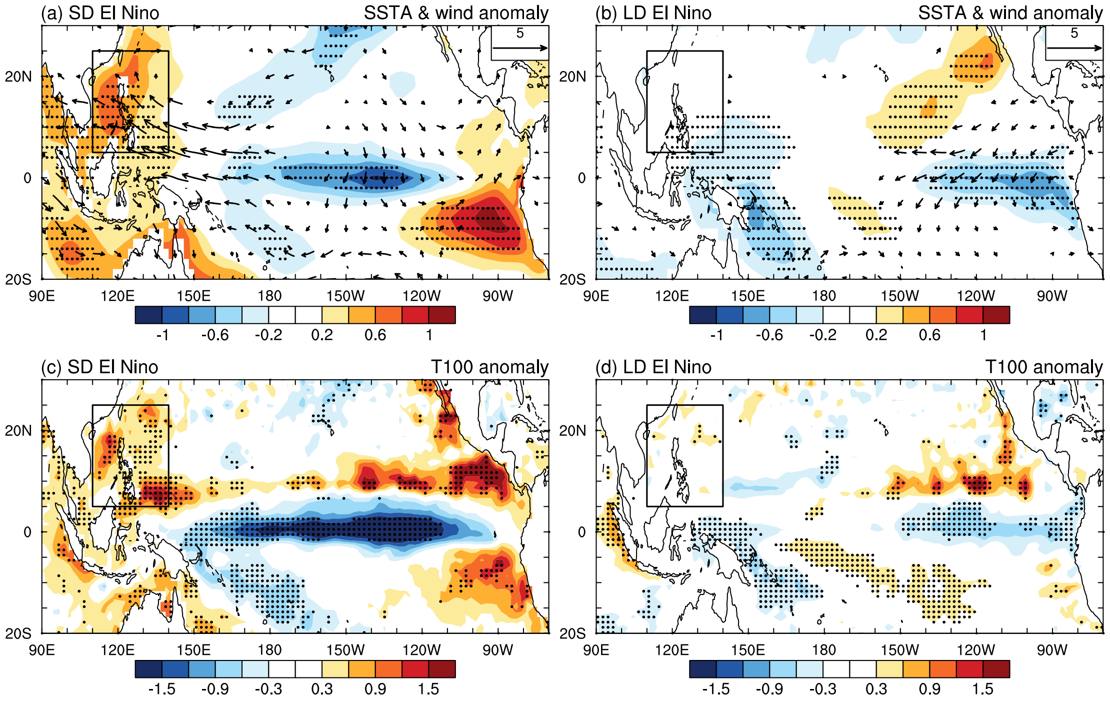

Supplementary Figure 23. Low-level wind and upper ocean thermal conditions during the short duration and long duration El Niño events Composite anomalies of (a) sea surface temperature (shading, units: K), and 850 hPa wind (vectors, units: m s^{-1}), and (c) averaged ocean temperature between 5–100 m (units: K) during the short duration El Niño events; (b, d) as in (a, c), but for the long duration El Niño events. The stippled areas indicate the values are statistically significant at the 95% confidence levels. Black boxes indicate the South China Sea and the western Philippine Sea region. Wind anomalies less than 0.2 m s^{-1} are not shown.

	Occurrence Longitude (degree)	TC Genesis Longitude (degree)	Occurrence Time (hr)		Duration Time (hr)	Frequency	Peak Intensity (knot)
			West	East			
SD El Niño	–8.04** (0.001)	–5.56 (0.108)	–20.94** (0.005)	43.69** (0.023)	4.52* (0.078)	–0.58 (0.281)	7.11 (0.315)
LD El Niño	1.23 (0.635)	0.61 (0.794)	–2.81 (0.887)	–2.16 (0.838)	3.09 (0.117)	1.32 (0.584)	5.59 (0.27)
All El Niño	–2.30 (0.237)	–1.74 (0.39)	–11.37 (0.307)	12.17 (0.212)	3.64** (0.024)	–0.19 (0.903)	6.17 (0.148)

Supplementary Table 1. Statistics of the rapid intensification related variables over western North Pacific Composite differences of the rapid intensification (RI) related mean occurrence longitude, tropical cyclone (TC) genesis longitude, occurrence time, duration time, frequency and peak intensity for short duration, long duration and all El Niño events with respect to those of neutral years. The positive (negative) Occurrence Longitude and RI TC Genesis Longitude anomalies indicate eastward (westward) migration of the TC RI occurrence position and RI TC genesis position, respectively. The positive (negative) Occurrence Time anomalies indicate the RIs occur later (earlier) than those in neutral years. The positive Duration Time anomalies indicate the RI processes last longer than those in neutral years. The positive (negative) Frequency anomalies indicate more (less) RI TCs occur than those in neutral years. The positive Peak Intensity anomalies indicate the peak intensity of RI TCs is stronger than those in neutral years. The values in the bracket are the P-values indicating the significance levels of the

composite difference. “West” and “East” in the RI occurrence time column indicate the values associated with the TCs formed over the west and east of 140°E, respectively. ** and * represent the 95% and 90% confidence levels, respectively.

	VWS	RH	TCHP	SST	T100
SD El Niño (N=20)	20 (1)	20 (1)	7 (3)	10 (2)	7 (3)
LD El Niño (N=25)	25 (1)	25 (1)	12 (2)	25 (1)	12 (2)
Neutral (N=220)	220 (1)	55 (4)	16 (14)	73 (3)	55 (4)

Supplementary Table 2. **Effective degrees of freedom of atmospheric and oceanic variables** Effective degrees of freedom (EDF) of the vertical wind shear (VWS), relative humidity (RH), TC heat potential (TCHP), sea surface temperature (SST), and ocean temperature averaged between 5–100 m (T100) for short duration El Niño, long duration El Niño and neutral years. N is the sample size, and values in the brackets indicate the decorrelation time scale.
